# Modulation of gut microbiota and intestinal immune response in gilthead seabream (*Sparus aurata*) by dietary bile salt supplementation

**DOI:** 10.3389/fmicb.2023.1123716

**Published:** 2023-04-24

**Authors:** Alberto Ruiz, Karl B. Andree, Dolors Furones, Paul G. Holhorea, Josep À. Calduch-Giner, Marc Viñas, Jaume Pérez-Sánchez, Enric Gisbert

**Affiliations:** ^1^Aquaculture Program, Institut de Recerca i Tecnologia Agroalimentàries (IRTA), Centre de La Ràpita, La Ràpita, Spain; ^2^Ph.D. Program in Aquaculture, Universitat de Barcelona, Barcelona, Spain; ^3^Nutrigenomics and Fish Growth Endocrinology Group, Institute of Aquaculture Torre de la Sal, Consejo Superior de Investigaciones Científicas (CSIC), Madrid, Castellón, Spain; ^4^Sustainability in Biosystems, Institut de Recerca i Tecnologia Agroalimentàries (IRTA) Torre Marimon, Barcelona, Spain

**Keywords:** fish gut microbiome, immune response, aquaculture, feed additive, bile salts, *Sparus aurata*, teleost, intestinal health

## Abstract

Given their role in lipid digestion, feed supplementation with bile salts could be an economic and sustainable solution to alterations in adiposity and intestinal inflammation generated by some strategies currently used in aquaculture. An important part of the metabolism of bile salts takes place in the intestine, where the microbiota transforms them into more toxic forms. Consequently, we aimed to evaluate the gut immune response and microbial populations in gilthead seabream (*Sparus aurata*) fed a diet supplemented with a blend of bile salts with proven background as a regulator of lipid metabolism and fat content. After the 90-day feeding trial, a differential modulation of the microbiota between the anterior and posterior intestine was observed. While in the anterior intestine the relative abundance of Desulfobacterota doubled, in the posterior intestine, the levels of Firmicutes increased and Proteobacteria, Actinobacteriota, and Campylobacterota were reduced when supplementing the diet with bile salts. Even so, only in the anterior intestine, there was a decrease in estimated richness (Chao1 and ACE indices) in presence of dietary bile salts. No significant differences were displayed in alpha (Shannon and Simpson indices) nor beta-diversity, showing that bile sales did not have a great impact on the intestinal microbiota. Regarding the gene expression profile in 2 h postprandial-fish, several changes were observed in the analyzed biomarkers of epithelial integrity, nutrient transport, mucus production, interleukins, cell markers, immunoglobulin production and pathogen recognition receptors. These results may indicate the development of an intestinal immune-protective status to tackle future threats. This work also suggests that this immune response is not only regulated by the presence of the dietary bile salts in the intestine, but also by the microbial populations that are in turn modulated by bile salts. After a fasting period of 2 days, the overall gene expression profile was stabilized with respect to fish fed the unsupplemented diet, indicating that the effect of bile salts was transient after short periods of fasting. On the balance, bile salts can be used as a dietary supplement to enhance *S. aurata* farming and production without compromising their intestinal health.

## 1. Introduction

According to estimates made by the FAO, aquaculture production will increase with respect to 2020 by 14% (24 million tons) in 2030, which would imply an increase in fish oil manufacturing of 13% [Food and Agriculture Organization of the United Nations (FAO), 2022]. The limited availability (depletion of resources and insufficient production) and high cost of fish oil have called into question the sustainability of the major source of omega-3 (n-3) fatty acids in aquafeeds. Under this scenario, the increasing production of plant-derived oils and their lower market prices in comparison to fish oil have made these alternative oil sources an attractive ingredient for fish feed manufacturers. Indeed, several studies have shown that the partial or total replacement of fish oil by plant-based oils do not significantly affect fish growth and feeding performance (Turchini et al., 2003; Ng et al., 2004; Fonseca-Madrigal et al., 2005; Drew et al., 2007). Nonetheless, most vegetable oils have deficiencies in n-3 long-chain highly unsaturated fatty acids (LC-HUFA) with respect to fish oil, which leads to alterations in the fatty acid profile, decreasing the concentration of docosahexaenoic acid (DHA) and eicosapentaenoic acid (EPA; Caballero et al., 2002; Turchini et al., 2003; Ng et al., 2004; Fonseca-Madrigal et al., 2005; Tocher et al., 2006; Drew et al., 2007). Furthermore, some physiological disorders such as a higher accumulation of lipid droplets on the hepatocytes and enterocytes have been observed under substitution of fish oil by plant-based oils (Olsen et al., 2000; Caballero et al., 2002). In the same way, although the replacement of fish oil by other animal fats in fish diets does not usually generate significant differences in fish performance, it may cause alterations in the fatty acid profile of the filet (Turchini et al., 2003; Subhadra et al., 2006; O’Neal and Kohler, 2008; Trushenski et al., 2011), thereby altering the expected nutritional content for the consumer. In addition, other nutritional alternatives, such as increasing the levels of dietary lipids or carbohydrates to spare the content of protein in the diet, may have a negative impact on growth and feeding performance, as well as modify the filet’s fatty acid profile and increase the accumulation of mesenteric fat (Rueda-Jasso et al., 2004; Du et al., 2006).

The above-mentioned studies are just examples of how the aquaculture industry is moving ever closer toward finding a cheaper and more sustainable strategy than the use of fish oil in aquafeeds, and the only wall that now separates the fish farmer from the use of the proposed alternatives is the potential deregulation of the fish lipid profile and problems in adiposity that they can entail. Following the path of the livestock industry (Lothong et al., 2016; Siyal et al., 2017), the increasing use of feed additives could be a functional solution to the above-mentioned problems as long as the selected feed additives promote lipid metabolism in a holistic manner. In this context, dietary bile salt (BS) supplementation may be a successful nutritional strategy due to their well-known role in the enhancement of lipid digestion and absorption, thanks to the activation of lipase and to the formation of micelles that allows emulsification of lipid aggregates (Romano et al., 2020). Furthermore, bile acids (BAs) can act as nutrient signaling hormones, regulating several biological processes by activating specific nuclear receptors, such as the farnesoid X receptor (FXR). Some of the mechanisms under regulation of FXR are metabolism of BSs, lipids, proteins, and carbohydrates, nutrient uptake, energy homeostasis, immunity, and, indirectly, the composition of the gut microbial communities, since they are shaped depending on the BS profile (Vavassori et al., 2009; Renga et al., 2013; Lickwar et al., 2017; Schubert et al., 2017; Chiang and Ferrell, 2022). In recent years, a considerable number of studies have been conducted to test the effects of dietary BS supplementation on fish performance, pointing to the possibility of their becoming a widely extended alternative within the aquafeed production industry. Addition of BSs to aquafeeds has not only successful results in terms of growth and feeding performance, but also enhances lipid catabolism and apparent lipid digestibility (Yamamoto et al., 2007; Iwashita et al., 2008; Gu et al., 2017; Jiang et al., 2018; Ding et al., 2020; Zhang et al., 2022; Ruiz et al., 2023). These actions provide benefits in terms of performance and generally translates into a reduction in lipid content of the body, reduction in fat storage deposits in the liver and in the intestine, as well as the strengthening of antioxidant defenses (Yamamoto et al., 2007; Iwashita et al., 2009; Jiang et al., 2018; Ding et al., 2020; Yin et al., 2021; Zhang et al., 2022; Ruiz et al., 2023).

Despite all the mentioned benefits for the physiology of fish and final quality of the product of dietary BS supplementation, there are not many studies focused on the effect of these molecules at an intestinal level. The importance of this organ lies in the fact that BS metabolism is not only regulated by hepatic FXR, but also by intestinal FXR, which has been suggested to be more sensitive to BAs (Houten et al., 2007) and to be the main regulator of BS metabolism via the classical pathway (Kong et al., 2012). Moreover, it has been proven that these molecules modulate the structure of the gut microbial communities, which metabolize primary BSs into secondary BSs. Although secondary BSs are more hydrophobic and thus presumably more toxic (Hofmann, 1999; Romano et al., 2020), the gut microbiota prevents their toxicity through generation of BS species that are different from those of the host (Schubert et al., 2017; Markandey et al., 2021). In addition, some studies have suggested that the intestinal microbiota plays a fundamental role in the proper development and functionality of the gut immune system (Broom and Kogut, 2018; Markandey et al., 2021). As mentioned above, BSs may also participate in the host immune response as mediated by FXR, which has a key role in the modulation of the intestinal immunity and maintenance of homeostasis (Vavassori et al., 2009; Renga et al., 2013). Due to the conservation of FXR-mediated pathways in fish, it would not be surprising that the effect of BSs reported in higher vertebrates dealing with the modulation of the intestinal microbiome and immunity could be extrapolated to fish. That has already been anticipated by the works of Yamamoto et al. (2007) and Iwashita et al. (2008, 2009), which demonstrated the anti-inflammatory effect of dietary BS supplementation on the intestine of rainbow trout (*Oncorhynchus mykiss*). Nevertheless, it should be noticed that at certain concentrations BSs can have an antimicrobial effect on some gut bacterial strains regarding several factors such the BS combination and dose, and environmental conditions like pH (Begley et al., 2005) and may cause a cytotoxic effect on the fish intestinal mucosa (Romano et al., 2020). The noxious effect of BSs in the intestine and its microbial communities is not only caused by their accumulation, but also by their contribution to the release of other substances that can be toxic if they accumulate, like bilirubin (Romano et al., 2020) and hydrogen sulfide (Schubert et al., 2017). This may lead to alterations in the metabolic functionality of the intestine, and in the production of pathogen-associated molecular patterns (PAMPs; Schubert et al., 2017). Under these premises, the nutritional assays testing BSs as feed additives performed by the aquaculture and, in general, livestock industry should not only focus on the animal performance but should also offer a holistic view of the health and condition of the studied holobiont (the organism and all of its associated microorganisms), including the organ which could be considered as the main target of BSs: the intestine.

In a previous work, we evaluated the effect of a blend of BSs (sodium cholate, sodium deoxycholate, and sodium taurocholate hydrate) on fish performance and studied the pathways underlying hepatic lipid metabolism in gilthead seabream (*Sparus aurata*) fed with a diet with high-saturated fat content. According to those results, fish displayed an enhanced growth performance and reduced levels of perivisceral, hepatic, and intestinal fat at a dietary BS inclusion level of 0.06% with respect to fish fed the unsupplemented diet (Ruiz et al., 2023). In this contribution, we moved a step forward by evaluating the microbial gut communities and intestinal immune status of *S. aurata* when supplementing the mentioned basal diet with the BS blend.

## 2. Materials and methods

### 2.1. Rearing conditions, feeding trial, and experimental diets

Juveniles of *S. aurata* (body weight, BW_i_ = 44.0 ± 4.2 g; standard length, SL = 12.13 ± 0.48 cm) were obtained from Piscicultura Marina Mediterranea SL (Valencia, Spain). Once at IRTA research facilities, fish (*N* = 240 individuals) were acclimated for 2 weeks and randomly distributed in 8 tanks of 450 L (30 fish per tank; density of 3 kg m^−3^) connected to a water recirculation system (IRTAmar™). Monitoring of water temperature (22.5 ± 0.5°C), dissolved oxygen (6.3 ± 0.2 mg L^−1^; OXI330, Crison Instruments, Spain), and pH (7.6 ± 0.01; pH meter 507, Crison Instruments) was carried out daily, and salinity (36‰; MASTER-20 T Hand-Held Refractometer, ATAGO Co. Ltd., Italy), nitrite (0.16 ± 0.1 mg NO_2_^−^ L^−1^), and ammonia (0.22 ± 0.08 mg NH_4_^+^ L^−1^) levels (HACH DR 900 Colorimeter, Hach Company, Spain) were measured weekly.

The trial lasted for 90 days, in which fish were fed twice a day in 12 servings of 5 min each by automatic feeders (Arvo-Tec T Drum 2000, Arvo-Tec Oy, Finland). Initial feeding rate was 3% and was regularly adjusted, as described in Salomón et al. (2020). Somatic growth was monthly monitored by netting all fish in each tank and their BW (g) and SL (cm) was measured. Once netted, fish were immediately anesthetized with 100 mg L^−1^ of buffered tricaine methanesulfonate (MS-222, Sigma-Aldrich, Spain).

Two experimental extruded diets (pellet size: 3 mm) were formulated and manufactured by Sparos Lda. (Portugal) as described by Ruiz et al. (2023). Diets were isoproteic (44.0% crude protein), isolipidic (18.0% crude fat), and isoenergetic (21.4 MJ kg^−1^) and only differed in their content of the BSs blend at an inclusion level of 0.06% (BS_0.06%_). The ingredients and proximate composition in dry form of the two experimental diets are shown in Table 1. The BS blend was composed of a 70% of sodium taurocholate hydrate (ref. 86,339, Sigma-Aldrich, United States) and a 30% of a powder BS mixture containing equal parts of sodium cholate and sodium deoxycholate (ref. 48,305, Sigma-Aldrich, United States).

**Table 1:** Formulation, proximate composition (in dry form) and fatty acid profile of the control diet and the basal diet supplemented with a blend of bile salts (BSs) at a dietary inclusion of 0.06% (BS_0.06%_).

Ingredients (%)	Control	BS_0.06%_
Fishmeal super prime	7.50	7.50
Fishmeal 60	5.00	5.00
Fish protein concentrate	2.00	2.00
Feather meal hydrolysate	5.00	5.00
Porcine blood meal	3.00	3.00
Poultry meal	15.00	15.00
AminoPro NT70—*C. glutamicum*	4.00	4.00
Corn gluten meal	8.00	8.00
Soybean meal 48	12.00	12.00
Sunflower meal	5.00	5.00
Wheat meal	10.31	10.31
Whole peas	5.00	5.00
Pea starch (raw)	2.40	2.40
Fish oil	3.02	3.02
Soybean oil	2.35	2.35
Poultry fat	8.04	8.04
Vitamin and mineral premix	1.00	1.00
Vitamin C35	0.05	0.05
Vitamin E50	0.02	0.02
Betaine HCl	0.20	0.20
Choline chloride 60	0.10	0.10
Antioxidant	0.20	0.20
Sodium propionate	0.10	0.10
Monoammonium phosphate	0.35	0.35
L-Tryptophan	0.15	0.15
DL-Methionine	0.20	0.20
Bile salts mix	-	0.06
Yttrium oxide	0.02	0.02
Proximate composition		
Crude protein, %	44.1 ± 0.05	44.0 ± 0.08
Crude fat, %	18.1 ± 0.04	18.2 ± 0.05
Gross energy, MJ kg^−1^	21.4 ± 1.11	21.5 ± 1.20
Fatty acid profile (% of total fatty acids)*		
Saturated fatty acids (SFAs)	27.19 ± 0.40	26.55 ± 0.06
Monounsaturated fatty acids (MUFAs)	36.61 ± 0.73	36.60 ± 0.22
n-6 Polyunsaturated fatty acids (n-6 PUFAs)	26.65 ± 0.06	27.11 ± 0.45
n-3 Polyunsaturated fatty acids (n-3 PUFAs)	9.55 ± 0.39	9.74 ± 0.09
Total PUFAs	36.20 ± 0.45	36.86 ± 0.36

* Fatty acid profile of experimental diets is detailed in Supplementary Table 1. The proximate and fatty acid composition of diets were analyzed in duplicate, and values are represented as mean ± SD.

### 2.2. Ethics statement

Animal procedures were performed according to the Spanish legislation (law 32/2007 and Royal Decree 1201/2015) and to the Guiding Principles for Biomedical Research Involving Animals (EU2010/63) and approved by the Ethical Committee of the Institute for Food and Agriculture Research and Technology (IRTA), which adopts “The European Code of Conduct for Research Integrity,” and by the Generalitat of Catalunya (CEEA 219/2020).

### 2.3. Sampling

After a fasting period of 48 h at the end of the trial, five fish per tank were randomly hand-netted, euthanized with an overdose of anesthetic MS-222 (300 mg L^−1^) and eviscerated. Then, a small segment (*ca.* 2 cm^2^) from the anterior intestine (AI) of two fish per tank (8 per treatment) was immersed in five volumes of RNAlater® (Sigma-Aldrich, United States), incubated overnight at 4°C, and stored at −80°C until RNA extraction in order to evaluate their gene expression profile, following the procedures of Ruiz et al. (2023). This region of the intestine was chosen because of its demonstrated immunological specialization as described in Vallejos-Vidal et al. (2022), and it was also made to coincide with the region sampled for histological analysis in the previous study of Ruiz et al. (2023). With the goal of studying the gut microbial communities, a 4 cm long section of AI just after the pyloric caeca and a section of *ca.* 4 cm from the posterior intestine (PI), from the anus backward, were dissected from the other three slaughtered fish (12 per treatment). The purpose of the fasting period was to avoid allochthonous microbiota, just targeting the autochthonous bacterial gut communities attached to intestinal mucus (Hao and Lee, 2004). The AI and PI segments of each fish were aseptically opened lengthwise, and their inner walls were separately scraped, insistently but gently with a round edge spatula, avoiding getting host smooth muscle and epithelia, to recover only mucosal bacteria. The scraped content of each segment (12 per dietary treatment in order to ensure statistical robustness) was immediately frozen at −80°C until DNA extraction. Anterior and posterior intestines were treated separately because of the differential digestion and absorption rate in each segment, being higher in the anterior region (Bakke et al., 2010), and because the metabolization of primary BSs into secondary BSs mainly takes place in the posterior part (Hofmann, 1999), which could cause a divergence of the observed microbiota between both regions.

To restore the non-fasting physiology of fish, the remaining animals in the tanks were fed for 3 days, and after 2 h from the last feeding (2 h postprandial-animals), two fish from each tank (8 per treatment) were randomly selected and their AI were dissected and stored at −80°C for RNA extraction as explained above, for assessing the temporal effect of the diets in the intestinal gene expression profile.

### 2.4. Intestinal gene expression profile

Following the manufacturer’s instructions of the QIAGEN RNeasy® Mini Kit (ref. 74,106, QIAGEN, Germany), RNA from AI was extracted and its concentration and purity were measured (NanoDrop-2000® spectrophotometer, Thermo Fisher Scientific, United States). The range of RNA concentrations was between 20 and 100 ng/μL, with A_260_/A_280_ absorbance ratios of 1.9–2.1. The integrity of RNA was also verified through an agarose gel electrophoresis. For cDNA synthesis, the High-Capacity cDNA Archive Kit (Applied Biosystems, United States) was used with an initial input of 500 ng of RNA. As a negative control, reactions without reverse transcriptase were run.

As described by Naya-Català et al. (2021), real-time quantitative PCR (qPCR) was performed with a CFX96 Connect™ Real-Time PCR Detection System (Bio-Rad, USA). Simultaneous profile of a panel of 44 genes was carried out by means of 96-well PCR array layouts, including biomarkers of epithelial integrity, nutrient transport, mucus production and innate and adaptative immunity (Table 2). Specific primer pair sequences are shown in Supplementary Table 2. Gene expression was calculated using the delta–delta Ct method (Livak and Schmittgen, 2001). The GeNorm software (M score = 0.21) was used for testing the gene expression stability of the gene *β-actin*, which was taken as an endogenous control in the normalization procedure. For multigene analysis, values were referenced to those of *hes1-b* of fish fed the control diet.

**Table 2:** PCR-array layout for gene expression profile of the intestine of gilthead seabream (*Sparus aurata*) fed experimental diets.

Function	Gene	Symbol	GenBank
Epithelial integrity	Proliferating cell nuclear antigen	*pcna*	KF857335
	Transcription factor HES-1-B	*hes1-b*	KF857344
	Krüppel-like factor 4	*klf4*	KF857346
	Claudin-12	*cldn12*	KF861992
	Claudin-15	*cldn15*	KF861993
	Cadherin-1	*cdh1*	KF861995
	Cadherin-17	*cdh17*	KF861996
	Tight junction protein ZO-1	*tjp1*	KF861994
	Desmoplakin	*dsp*	KF861999
	Gap junction Cx32.2 protein	*cx32.2*	KF862000
	Coxsackievirus and adenovirus receptor homolog	*cxadr*	KF861998
Nutrient transport	Intestinal-type alkaline phosphatase	*alpi*	KF857309
	Liver type fatty acid-binding protein	*fabp1*	KF857311
	Intestinal fatty acid-binding protein	*fabp2*	KF857310
	Ileal fatty acid-binding protein	*fabp6*	KF857312
Mucus production	Mucin 2	*muc2*	JQ277710
	Mucin 13	*muc13*	JQ277713
Interleukins	Tumor necrosis factor-alpha	*tnf-α*	AJ413189
	Interleukin-1 beta	*il-1β*	AJ419178
	Interleukin-6	*il-6*	EU244588
	Interleukin-7	*il-7*	JX976618
	Interleukin-8	*il-8*	JX976619
	Interleukin-10	*il-10*	JX976621
	Interleukin-12 subunit beta	*il-12β*	JX976624
	Interleukin-15	*il-15*	JX976625
	Interleukin-34	*il-34*	JX976629
Cell markers	Cluster of differentiation 4–1	*cd4-1*	AM489485
	Cluster of differentiation 8 beta	*cd8b*	KX231275
	C-C chemokine receptor type 3	*ccr3*	KF857317
	C-C chemokine receptor type 9	*ccr9*	KF857318
	C-C chemokine receptor type 11	*ccr11*	KF857319
	C-C chemokine CK8 / C-C motif chemokine 20	*ck8/ccl20*	GU181393
	Macrophage colony-stimulating factor 1 receptor 1	*csf1r1*	AM050293
Ig production	Immunoglobulin M	*igm*	JQ811851
	Immunoglobulin T membrane-bound form	*igt-m*	KX599201
Pathogen-associated microbial pattern (PAMP)	Galectin-1	*lgals1*	KF862003
	Galectin-8	*lgals8*	KF862004
	Toll-like receptor 2	*tlr2*	KF857323
	Toll-like receptor 5	*tlr5*	KF857324
	Toll-like receptor 9	*tlr9*	AY751797
	CD209 antigen-like protein D	*cd209d*	KF857327
	CD302 antigen	*cd302*	KF857328
	Macrophage mannose receptor 1	*mrc1*	KF857326
	Fucolectin	*fcl*	KF857331

### 2.5. Gut microbial analyses

Extraction of the DNA from *ca.* 250 mg of the scraped product of 12 AI and 12 PI from each dietary group (three per tank) was carried out with the DNeasy PowerSoil Pro Kit (ref. 47016, QIAGEN, Germany), following the manufacturer’s recommendations. Prior to performing extractions, a previous step of bead-beating for sample homogenization and cell lysis (BioSpec Mini-BeadBeater-8, BioSpec Products, United States) was performed. DNA concentration and purity were measured in a NanoDrop-2000® spectrophotometer (Thermo Fisher Scientific, United States). The values of A_260_/A_280_ absorbance ratios were higher than 1.85, and DNA concentrations ranged up to 500 ng μL^−1^.

The V3–V4 region of the 16S rRNA gene was amplified using the bacterial universal primers 341F (5′-CCTACGGGNGGCWGCAG-3′) and 805R (5′-GACTACHVGGGTATCTAATCC-3′; Klindworth et al., 2013) under the following conditions: 30 s at 98°C, followed by 30 cycles of 10 s at 98°C, 30 s at 55°C and 30 s at 72°C, and a final elongation step of 2 min at 72°C. Library generation was performed according to 16S Metagenomic Sequencing Library Preparation guide (Illumina, 2013) and pair-end 2 × 300 bp sequencing was carried out by means of Illumina-MiSeq platform. Two samples were excluded in the process due to low amplified product concentration. Raw sequencing data were deposited in the Sequence Read Archive (SRA) of NCBI under BioProject accession number PRJNA915342.

Bioinformatic analyses were performed as follows: forward and reverse primers were removed from the raw paired-ended reads by means of the Cutadapt tool in QIIME2 Software (version 2021.11). Then, data were exported to RStudio (version 4.1.2) and processed using the R package *dada2* (Callahan et al., 2016). Forward and reverse read qualities were assessed individually by sample, and by total average (Supplementary Figure 1). An individual and average quality threshold of 28 was established, excluding the reads with a lower Phred score, and those with higher expected error than 2. Then, the paired-ended reads were assembled into contigs, removing the ones with an overlap length < 12 nucleotides or with more than 0 mismatches in the overlap region. A total of 3.3% of the sequences were identified as chimeras and discarded from analysis. Then, SILVA (v138.1) was used as the reference database for bacterial taxonomy classification of contigs into amplicon sequence variants (ASVs), establishing a bootstrapping cut-off of 80% to be considered as a reliable assignment (Smith et al., 2020); otherwise, they were classified as unassigned. Those ASVs with a total sum < 3 sequences (singletons and doubletons) were removed. In brief, from 45 samples a total of 2,900,687 sequences clustering into 14,507 ASVs were generated. According to rarefaction curves (obtained with *vegan* library; Supplementary Figure 2), sample depths were rarefied to 50,000 reads and normalized by total sum scaling (TSS) following the recommendations of McKnight et al. (2019). After rarefaction, a total of 2,250,000 sequences clustering into 14,500 AVSs were obtained. Alpha-diversity was approached by Chao1 and ACE indices for estimating richness and by Shannon and Simpson indices for assessing diversity. The ACE index takes into account rare ASVs (“rare” defined as those with fewer than 10 reads per sample; Kim et al., 2017).

### 2.6. Statistical analyses

Data on gene expression were analyzed by Student’s *t*-test (*p* < 0.05). A Shapiro–Wilk test was used for verifying normality of the data and the Holm-Sidak *post hoc* test for multiple comparisons among groups. Analysis of the interaction between the diets and nutritional status was evaluated with a two-way ANOVA and a Holm-Sidak post-test. To study the separation among dietary groups and nutritional status, supervised partial least squares-discriminant analysis (PLS-DA) and hierarchical clustering of statistically significant genes (*p* < 0.1) were sequentially applied using EZinfo (v.3.0, Umetrics, Sweden) and the R package *ggplot2*, respectively. Hotelling’s *T*^2^ statistic was calculated with the multivariate software package EZinfo and points above the 95% confidence limit for *T*^2^ were considered as outliers and discarded. The quality of the PLS-DA model was evaluated by the parameters R2Y(*cum*) and Q2(*cum*), which indicate the fit and prediction ability, respectively. To assess whether the supervised model was being over-fitted, a validation test consisting of 500 random permutations was performed using the *opls* function from the *ropls* R package.

Significant differences in alpha-diversity among groups (*p* < 0.05) were determined by Kruskal—Wallis one-way analysis of variance, followed by Dunn’s post-test. Beta-diversity was calculated as Bray–Curtis dissimilarity (Bray and Curtis, 1957) and represented in a principal coordinate analysis (PCoA). To check significant differences in beta-diversity, permutational multivariate analyses of variance (PERMANOVA) were performed (*p* < 0.05). Differential abundances between groups in phyla and genera with relative abundances >1% were calculated with the method *Metastats*, which includes adjustment of *p* value by false discovery rate (FDR; White et al., 2009). All the described microbial statistical analyses were executed with the R package *microeco* (Liu et al., 2021), which was used together with *ggplot2* for generation of figures.

## 3. Results

Results in terms of growth performance and feed efficiency are presented elsewhere (Ruiz et al., 2023). In brief, the supplementation of a blend of BSs promoted somatic growth and fish fed the BS_0.06%_ diet grew more (221.21 ± 3.10 g) than those fed the control diet (215.80 ± 1.06 g; *p* < 0.05). However, no differences in feed conversion ratio (FCR) values were found between both diets, with values ranging from 1.21 ± 0.05 in the control group to 1.19 ± 0.05 in the BS_0.06%_ diet (*p* > 0.05).

### 3.1. Alpha and beta-diversity of gut microbiota

**Figure 1:**
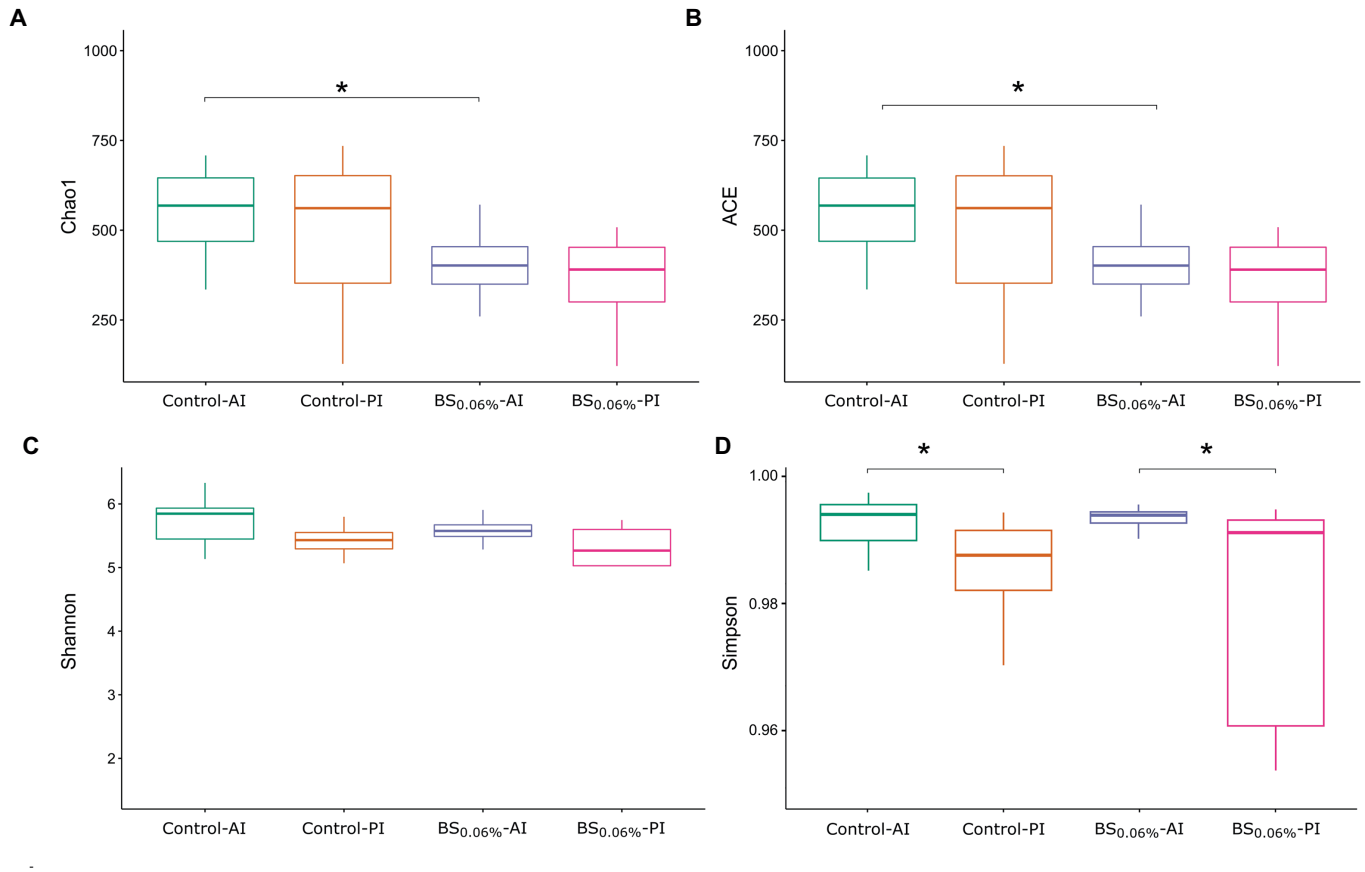
Box plots representing the minimum, maximum and the median of the sample values obtained from the richness estimators Chao1 **(A)** and ACE **(B)**, and diversity indices Shannon **(C)** and Simpson **(D)**. The asterisks represent the significant differences between dietary condition or intestinal region of gilthead seabream (*Sparus aurata*; Kruskal—Wallis, Dunn’s post-test, *P* < 0.05). Experimental groups (*n* = 12 fish per group): anterior (Control-AI) and posterior intestine (Control-PI) of *S. aurata* fed the control diet, and anterior (BS_0.06%_-AI) and posterior intestine (BS_0.06%_-PI) of *S. aurata* fed the BS_0.06%_ diet.

Figure 1 shows the estimated richness and diversity (alpha-diversity metrics) of gut microbial communities in the AI and PI regions in both experimental groups (Kruskal–Wallis, followed by Dunn’s test). Considering Chao1 and ACE indices, the addition of BSs to the diet generated a reduction in species richness in the AI in *S. aurata* (Figures 1A,B; *p* < 0.05), while no divergences in diversity were found (Figures 1C,D; *p* > 0.05). On the other hand, although no differences in richness were found between the AI and the PI (Figures 1A,B), Simpson’s diversity index experienced a significant decrease in the PI with respect to the AI in fish fed both diets (Figure 1D; Supplementary Table 3).

Significant differences in beta-diversity were found among the four experimental groups (PERMANOVA, *F* = 1.234, *R^2^* = 0.083, *p* = 0.024). However, no variation in beta-diversity was registered when comparing dietary condition or region of intestine by pairwise PERMANOVA (*p* > 0.05; see PCoA in Supplementary Figure 3). In fact, the only significant differences observed were between Control-PI and BS_0.06%_-AI samples (PERMANOVA, *R^2^* = 0.065, *p* = 0.037). The tank variable was verified as an insignificant effect for beta-diversity results (PERMANOVA, *F* = 1.1708, *R^2^* = 0.026, *p* = 0.114).

### 3.2. Gut microbiota composition

**Figure 2:**
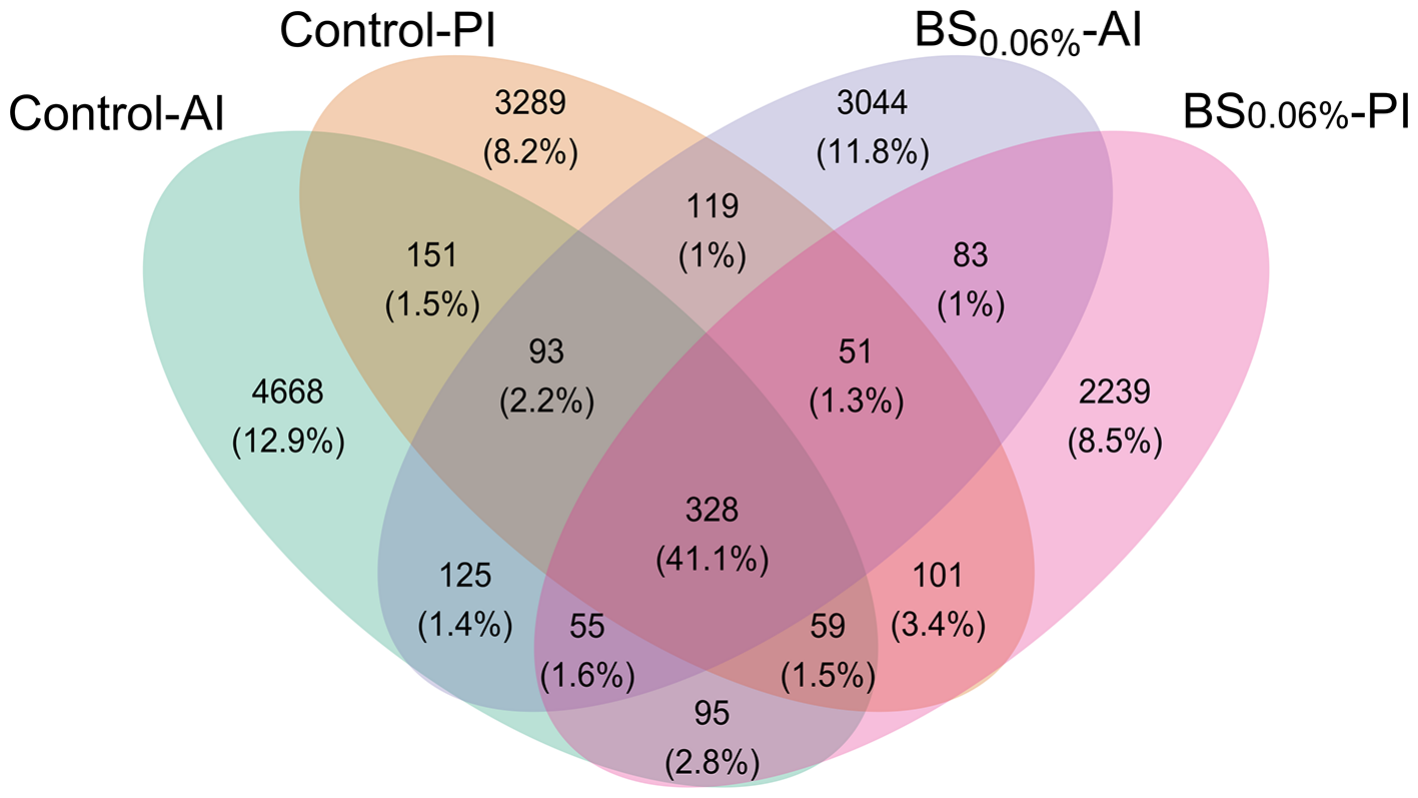
Venn diagram plotting the number and relative abundances (%) of ASVs that are unique or common among experimental groups. Experimental groups (*n* = 12 fish per group): anterior (Control-AI) and posterior intestine (Control-PI) of gilthead seabream (*Sparus aurata*) fed the control diet, and anterior (BS_0.06%_-AI) and posterior intestine (BS_0.06%_-PI) of *S. aurata* fed a basal diet supplemented with a blend of bile salts at a dietary inclusion level of 0.06% (BS_0.06%_).

After rarefaction (50,000 reads per sample), a total of 14,500 ASVs were obtained. Among them, 5,574 ASVs (65.0% of total microbiota composition) were found in the AI (Control-AI), and 4,191 (60.2%) in the PI (Control-PI) of fish fed the control diet; while in fish fed the BS_0.06%_ diet, 3,898 ASVs (61.4%) appeared in the AI (BS_0.06%_-AI) and 3,011 (61.2%) in the PI (BS_0.06%_-PI; Figure 2). Among them, 328 ASVs were common to the four groups (41.1%), whereas 4,668 ASVs (12.9%) were exclusive to the Control-AI; 3,289 ASVs (8.2%) were exclusive to the Control-PI; and 3,044 ASVs (11.8%) were exclusive to the BS_0.06%_-AI; and 2,239 (8.5%) to the BS_0.06%_-PI.

**Figure 3:**
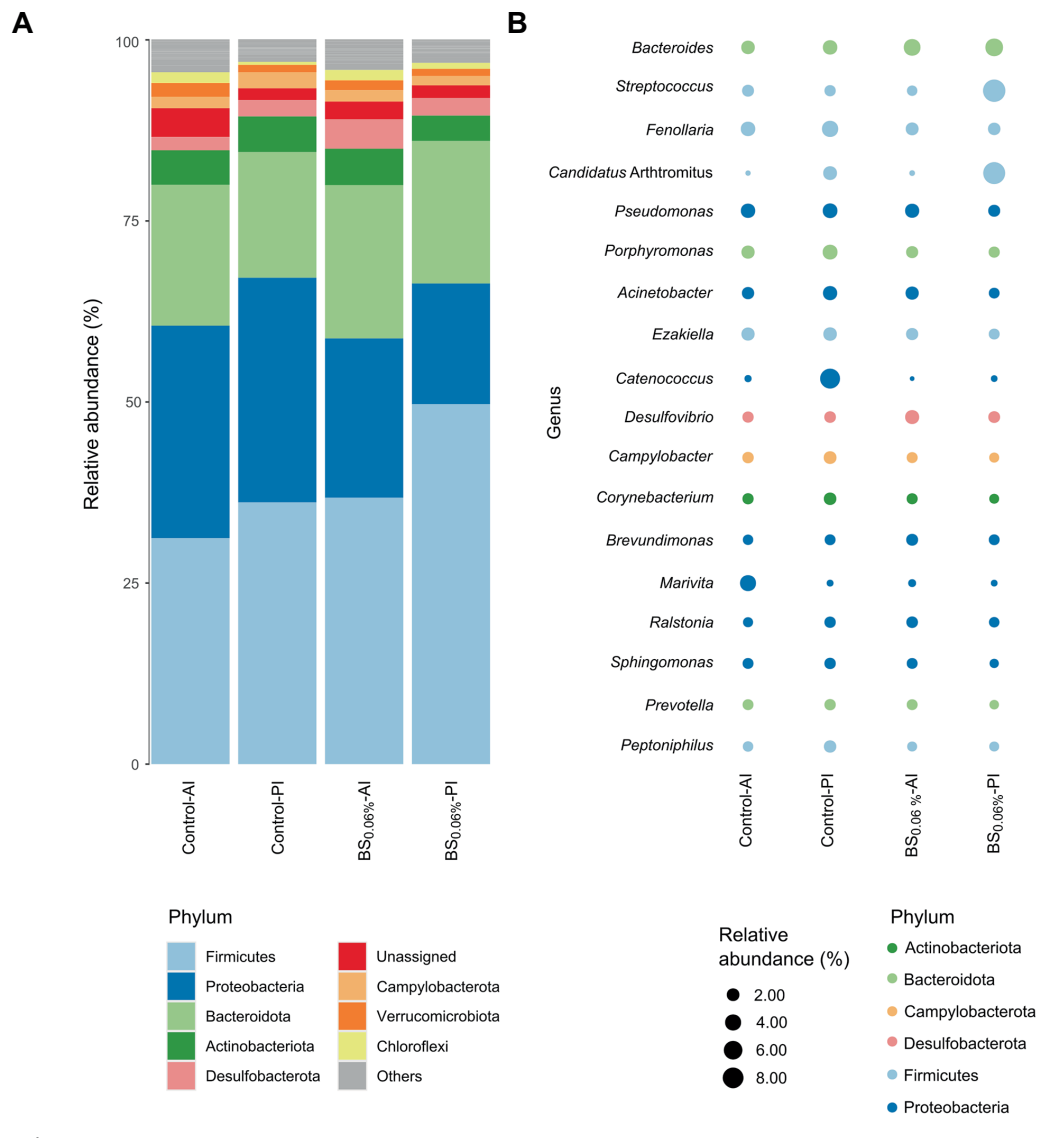
Relative abundances of gut bacterial taxa from gilthead seabream (*Sparus aurata*). Data are expressed at phylum **(A)** and genus **(B)** levels (excluding unassigned genera). Taxa appearance in the figures is in order of decreasing abundance (from bottom to top in the bar graph, and inversely in the bubble plot). Taxa with an abundance < 1% are classified as others in the bar graph and not represented in the bubble plot. Experimental groups (*n* = 12 fish per group): anterior (Control-AI) and posterior intestine (Control-PI) of *S. aurata* fed the control diet, and anterior (BS_0.06%_-AI) and posterior intestine (BS_0.06%_-PI) of *S. aurata* fed a basal diet supplemented with a blend of bile salts at a dietary inclusion level of 0.06% (BS_0.06%_).

The ASVs were further classified by phylum (Figure 3A) and genus (Figure 3B). The most abundant phylum was Firmicutes (31.2%–49.7%), followed by Proteobacteria (16.6%–31%), Bacteroidota (17.3%–21.2%), Actinobacteriota (3.5%–5.0%), Desulfobacterota (1.9%–4.1%), Campylobacterota (1.3%–2.2%), Verrucomicrobiota (1.0%–1.9%), and Chloroflexi (0.4%–1.5%). From the most abundant genera (samples’ average abundance > 1%; Figure 3B), seven of them belonged to the phylum Proteobacteria (*Pseudomonas*, *Acinetobacter*, *Catenococcus*, *Brevundimonas*, *Marivita*, *Ralstonia*, and *Sphingomonas*), five to Firmicutes (*Streptococcus*, *Fenollaria*, *Candidatus* Arthromitus, *Ezakiella*, and *Peptoniphilus*), three to Bacteroidota (*Bacteroides*, *Porphyromonas*, and *Prevotella*), one to Desulfobacterota (*Desulfovibrio*), one to Campylobacterota (*Campylobacter*), and one to Actinobacteriota (*Corynebacterium*).

When comparing the control to the BS_0.06%_ diet, significant differences were found among phyla (Supplementary Table 4). In particular, in fish fed with the BS_0.06%_ diet, Desulfobacterota abundance increased 2.2 times in the AI (*p* < 0.05; BS_0.06%_-AI vs. Control-AI), while in the PI, Firmicutes increased up to 1.4 times and Proteobacteria showed a decrease of 1.9 times (*p* < 0.05; BS_0.06%_-PI vs. Control-PI). To a lesser extent (*p* < 0.1), a reduction in Actinobacteriota and Campylobacterota abundances was also found in the PI of *S. aurata* fed the BS_0.06%_ diet (BS_0.06%_-PI) with respect to those fed the control diet (Control-PI).

At the genus level, the AI of fish fed the BS_0.06%_ diet presented higher abundances of *Bacteroides*, *Desulfovibrio*, *Brevundimonas*, and *Ralstonia* (*p* < 0.05), and an apparent slight decrease of *Streptococcus* (*p* < 0.1) was also registered with respect to fish fed the control diet (Supplementary Table 5; BS_0.06%_-AI vs. Control-AI). Otherwise, a significant reduction was found in *Porphyromonas*, *Campylobacter*, *Corynebacterium*, *Sphingomonas*, *Peptoniphilus*, and, to a lesser degree (*p* < 0.1), of *Fenollaria*, *Acinetobacter*, *Ezakiella*, and *Prevotella* in the PI of fish fed the BS-supplemented diet with respect to the control group (BS_0.06%_-PI vs. Control-PI).

### 3.3. Gene expression profile

All genes included in the PCR-array were found at detectable levels, as shown in Supplementary Table 6. In 2 h postprandial-animals, the relative expression of *cdh17*, *lgals8* (*p* < 0.05), and, especially, *alpi* (*p* < 0.01) was down-regulated, while *cd4-1*, *ccr9*, *ck8*/*ccl20* and *lgals1* were up-regulated (*p* < 0.05) in *S. aurata* fed the BS_0.06%_ diet with respect to those fed the control diet. To a lesser extent (*p* < 0.1), there was also a decrease in gene expression of *pcna*, *cx32.2*, *muc13*, and *tlr9*, and an increase in expression of *fabp6*, *il-8*, *ccr3*, *igt-m*, and *mrc1* in comparison with the control group. Summarizing, the addition of BSs to the diet in 2 h postprandial-fish induced an altered expression of some of the measured genes related to pathogen-associated microbial pattern (4), cell markers (4), and markers of epithelial integrity (3), among others. On the other hand, in 48 h fasted-animals the only significant differences in gene expression were the up-regulation of *cldn15* and *cxadr*, and the down-regulation of *cd8b* with respect to the control group (*p* < 0.05; Supplementary Table 6).

**Figure 4:**
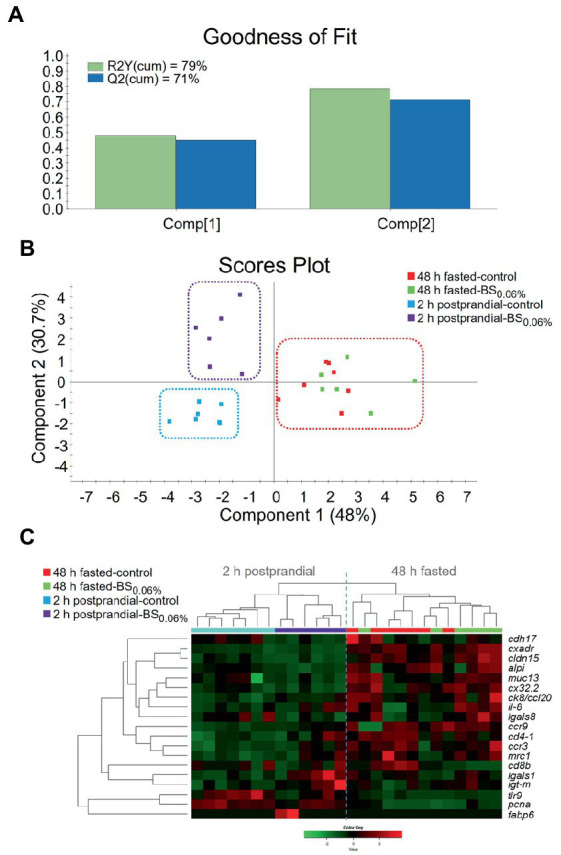
**(A)** Graphical representation of the goodness of fit of the PLS-DA model. **(B)** Scores plot for two-dimensional PLS-DA representing sample distribution between the two components of the model on the basis of their intestinal expression profiles based on statistically significant genes (*p* < 0.1). **(C)** Heatmap plotting hierarchical clustering among statistically significant genes (*p* < 0.1) on the basis of their expression pattern (Color Key scale) allowing to discriminate for sample distribution. Samples were obtained from anterior intestine (AI) of 48 h fasted- and 2 h postprandial-gilthead seabream (*Sparus aurata*; *n* = 8 per dietary treatment) that were fed the control and the experimental diet supplemented with a blend of bile salts at a dietary inclusion level of 0.06% (BS_0.06%_).

For evaluating differences in the expression profile of the AI, a two-component PLS-DA model was constructed, with a R2Y(*cum*) of 79% and a Q2(*cum*) of 71% (Figure 4A). The fit of the model was validated by a permutation test (Supplementary Figure 4). The first component of the PLS-DA (48.0% explained variance) clustered fish separately by their feeding condition (48 h fasting vs. 2 h postprandial), whereas the second component (30.7% explained variance) separated 2 h postprandial-animals from both dietary groups (Figure 4B). On the other hand, the PLS-DA showed that in 48 h fasted-animals, there was not a divergent distribution between both dietary groups. Results from the PLS-DA were supported by the expression pattern shown in the heatmap (Figure 4C), which grouped the 2 h postprandial-fish fed the control diet separately from those fed the BS_0.06%_ diet, while the two experimental groups of 48 h fasted-fish clustered together.

## 4. Discussion

### 4.1. Effect of dietary bile salt supplementation on intestinal microbiota

Under current experimental conditions, Firmicutes, Proteobacteria, Bacteroidota, and Actinobacteriota were the most abundant phyla found in the gut of *S. aurata*. These results were in agreement with previous nutritional studies in the same species (Tapia-Paniagua et al., 2020; Naya-Català et al., 2021). However, the dietary supplementation of BSs resulted in changes in the microbial richness of the intestine in *S. aurata* among dietary treatments. In this sense, a reduction in the observed (number of ASVs) and estimated richness (Chao1 and ACE indices) of bacterial communities was observed in the AI of fish fed the BS_0.06%_ diet with respect to those fed the control diet, even though such changes were not coupled to variations in bacterial diversity (Shannon and Simpson indices) among dietary groups. Previous studies conducted in fishes have reported that dietary BSs are capable of changing gut microbiota, although depending on the study considered, different changes in bacterial richness and diversity have been reported. In particular, diets supplemented with BSs resulted in differences in the bacterial community richness (Chao1 and ACE indices) in Chinese perch (*Siniperca chuatsi*; Zhang et al., 2022) and *S. aurata* (present results), whereas other studies have reported that dietary BSs only affected bacterial diversity (Shannon and Simpson indices) like in grass carp (*Ctenopharyngodon idella*) and tongue sole (*Cynoglossus semilaevis*; Xiong et al., 2018; Li et al., 2021). Such changes in bacterial richness and diversity among studies may be attributed to different blends and levels of tested BSs, as well as different basal diet formulations, fish husbandry, and physiological conditions.

Most relevant changes in gut microbiota derived from dietary BS supplementation were found in the PI, results that may be attributed to the fact that this region of the digestive tract is where primary BSs are metabolized by the microbiota into secondary BSs, which are more hydrophobic and noxious to bacteria (Romano et al., 2020). In our study, there was a significant increase in the relative abundance of Firmicutes coupled to a decrease in Proteobacteria and Actinobacteriota in the PI of fish fed the BS_0.06%_ diet. Such increase in Firmicutes relative abundance may be related to the higher tolerance and capacity of metabolizing BSs by this phylum of bacteria (Islam et al., 2011; Ridlon et al., 2014). When comparing the composition of the microbial communities at the genus level, there were also some remarkable variations between both dietary treatments. We found an increase in *Bacteroides* as it has been described in *C. idella* fed BS-supplemented diets (Xiong et al., 2018; Zhou et al., 2018), since they may use dietary BSs as substrates for their growth. In particular, *Bacteroides* is a Gram-negative group of bacteria that possess several enzymatic activities involved in the metabolization of primary BSs into secondary BSs (Kawamoto et al., 1989; Chattopadhyay et al., 2022), as well as the capacity to promote BA 7α-dehydroxylase activity in Gram-positive bacterial strains (Hirano and Masuda, 1982). Furthermore, the production of short chain fatty acids by this genus has been suggested to prevent obesity and promote lipolysis in some mammals (Al-Lahham et al., 2010; Li et al., 2013). Although this deserves further investigation in fish, the potential role of *Bacteroides* in lipolysis might explain the reduction in the perivisceral fat levels found in *S. aurata* (Ruiz et al., 2023) and in *C. idella* (Zhou et al., 2018) fed BS-supplemented diets. Furthermore, the increase in the relative abundance of the genus *Desulfovibrio* may be attributed to its capacity of using BA metabolites; in particular, the members of this Gram-negative genus are able to metabolize taurine released from deconjugated BAs, which, similarly to *Bacteroides*, may promote the growth of bacteria able to transform BAs by 7α-dehydroxylation (Hu et al., 2022). Additionally, the growth of *Desulfovibrio* may also be explained by the effect of cholic and deoxycholic acids on this genus (Chattopadhyay et al., 2022). In higher vertebrates, the increase in the relative abundance of *Desulfovibrio* has also been associated to improved health (Chen et al., 2021), findings that seemed to be in line with our previous results in *S. aurata* fed the BS-supplemented diet that showed a better condition in terms of growth and somatic indices, as well as in the levels of fat deposits in the visceral cavity and digestive organs (Ruiz et al., 2023).

While *Bacteroides* and *Desulfovibrio* are among the most reported anaerobic bacteria found in the gastrointestinal tract of marine fish (Romero et al., 2014), the aerobic genus *Brevundimonas* has not been so well studied in fish. In the present study, the increase in the levels of *Brevundimonas* in the gut of fish fed the BS_0.06%_ diet may be attributed to its role in the metabolization of BSs through 7α-hydroxysteroid dehydrogenase (7α-HSD) activity, playing an important role in secondary BA formation (Chattopadhyay et al., 2022). In addition, current results showed that using a BS-supplemented diet led to a reduction in the levels of some bacterial genera (*Acinetobacter*, *Corynebacterium*, *Peptoniphilus*, *Streptococcus*, *Sphingomonas*, *Porphyromonas*, and *Prevotella*), which are generally considered as commensal species in the gastrointestinal tract of *S. aurata* (Estruch et al., 2015; Naya-Català et al., 2021) and other fish species (Romero et al., 2014). However, as some species of the above-mentioned genera may also be potential emergent fish pathogens (Baya et al., 1992; Weinstein et al., 1997; Kozińska et al., 2014; Malick et al., 2020), the overall effect of this dietary strategy cannot be properly assessed unless further characterization of the microbial community at the species level is conducted. The above-mentioned changes in bacterial abundance may be attributed to their capacity in deconjugation and dehydroxylation of BAs (Schubert et al., 2017) as well as to the noxious effect of BAs, especially secondary BAs, on some species (Hofmann, 1999; Romano et al., 2020). Moreover, the absence of differences in beta-diversity hints that the supplementation of a high saturated fat diet with BSs does not pose any risk for dysbiosis of the fish gut microbiota.

### 4.2. Effect of dietary bile salt supplementation on intestinal gene expression profile

Different transcriptomic responses were observed when comparing both 48 h of fasting vs. 2 h postprandial samples. When looking at PLS-DA results, the control vs. BS_0.06%_ comparison showed different patterns of gene expression in fed fish (2 h postprandial-group), whereas this pattern disappeared when considering fish fasted for 48 h (Supplementary Table 6). That may indicate that the effect of BS_0.06%_ was transient and reversible after short fasting periods as it was previously postulated (Ruiz et al., 2023).

Only three genes (*cldn15*, *cxadr*, and *cd8b*) were found to be differentially expressed in fish fasted for 48 h. The tight junction protein claudin-15 (CLDN15) regulates Na^+^ homeostasis and epithelial permeability for cations (Tamura et al., 2011; Wada et al., 2013). In addition, the uptake of BAs into enterocytes takes place by a Na^+^-dependent transporter (Keating and Keely, 2009), which is under control of intestinal FXR (Sinha et al., 2008). Thus, the up-regulation of *cldn15* agrees with the up-regulation of coxsackievirus and adenovirus receptor homolog (*cxadr*), another tight junction gene involved in the regulation of epithelial permeability and tissue homeostasis (Raschperger et al., 2006). In addition to their regulation by dietary BSs, the above-mentioned genes may also be differentially regulated as a compensatory mechanism to the fasting period to which animals were exposed before sampling (Wada et al., 2013). The third gene differentially expressed between both dietary groups after 48 h of fasting was the *cd8b*. The cluster of differentiation 8 (CD8) is a co-receptor and signal transduction molecule, expressed on the surface of CD8^+^ T cells, playing an important regulatory role in immune responses and has antibacterial activity (Nakanishi et al., 2015). Thus, the reduction in *cd8b* expression might improve gut mucosal condition generated by the above-mentioned changes of bacterial abundance at the genus level rather than a depressed immune response.

When considering the effects of diets supplemented with BSs in 2 h postprandial-animals, we found a completely different scenario with 27% of the analyzed genes differentially expressed. The fish intestine is a complex multifunctional organ, responsible for feed digestion, nutrient absorption, water and electrolyte homeostasis, nutrient metabolism, and immunity (Buddington et al., 1997). This organ is also involved in BS metabolism through the gut-liver axis since the intestinal FXR is involved in the synthesis of BA in the liver (Romano et al., 2020). Thus, we found a differential expression pattern of some biomarkers involved in nutrient transport and absorption, such as the up-regulation of the fatty acid-binding protein (*fabp6*) and the down-regulation of intestinal-type alkaline phosphatase (*alpi*) in fish fed the BS-supplemented diet. Particularly, FABP6 is commonly regarded as a BA binding protein found in the distal portion of the intestine and is involved in the efficient apical to basolateral transport of conjugated BAs in enterocytes; thus, playing an important role in BA homeostasis (Praslickova et al., 2012). Such change in *fabp6* might be explained by changes in the BA profile in *S. aurata* fed the BS_0.06%_ (Ruiz et al., 2023), as well as by FXR (Lickwar et al., 2017), as it has recently been demonstrated in zebrafish (*Brachydanio rerio*; Wen et al., 2021). On the other hand, ALPI is located in the enterocyte brush border and participates in nutrient absorption, the maintenance of the gut barrier function, and modulation of gut microbiota (Lallès, 2020). Significantly, ALPI promotes an anti-inflammatory immune response through dephosphorylation of lipopolysaccharides (LPSs) from the outer membrane of Gram-negative bacteria, and its deficiency has been correlated with intestinal inflammation (Rader, 2017). However, under the present conditions no signs of enteric inflammation were found in fish fed the BS_0.06%_ diet (Ruiz et al., 2023); thus, the down-regulation of *alpi* in fish fed the BS_0.06%_ diet might be due to a reduced bacterial richness as indicated by microbial analyses (number of ASVs and, Chao1 and ACE indices).

The rest of the analyzed intestinal biomarkers that were also differentially expressed are mainly involved in epithelial integrity and immunity (*pcna*, *cdh17*, *cx32.2*, *muc13*, *il-8*, *cd4-1*, *ccr3*, *ccr9*, *ck8/ccl20*, *igt-m*, *lgals1*, *lgals8*, *tlr9*, and *mrc1*). Although there are not many studies in this field, some recent studies are beginning to elucidate the role of BSs in the intestine of fish (Jin et al., 2019; Peng et al., 2019; Yin et al., 2021). As Foey and Picchietti (2014) highlighted, the mucosal layer is the first line of defense of the fish immune system, which acts as a physical and chemical immune barrier, and consists of the mucus and its commensal bacteria that overlay the epithelial cells lining the gut with associated lymphoid tissue. In a previous nutritional trial of dietary BSs (Ruiz et al., 2023), the concentration of BSs used in the current work was identified as being effective at inducing an effect on body fat content while remaining within concentrations that are not toxic for the animal (Keating and Keely, 2009; Romano et al., 2020); and considering the transcriptomic results for biomarkers of gut epithelial integrity, we may state that this goal was achieved. In particular, the down-regulation of proliferating cell nuclear antigen (*pcna*) may suggest a lower epithelial turnover rate and consequently, an ameliorated health condition of enterocytes (Gisbert et al., 2017; Naya-Català et al., 2021). Lower epithelial cell turnover rate may be in line with the observed lower gene expression of the adherens- and gap-type junction proteins, cadherin-17 (*cdh17*) and gap junction Cx32.2 protein (*cx32.2*), respectively. The expression of the rest of the biomarkers related to epithelial integrity did not change among fish fed the control and the BS_0.06%_ diet. A high concentration of BSs in the intestine can cause a loss of the tight junctions between epithelial cells, leading to an increase in mucosal permeability and cell death (Keating and Keely, 2009), so the absence of differences in the expression of tight junction proteins confirmed that the gut barrier function was maintained at the concentration of BSs tested. In addition, the decrease in expression of mucin 13 (*muc13*) in intestinal cells could be correlated to a lower mucus turnover in response to a reduction of certain bacterial genera (Pérez et al., 2010).

Current data also indicated that the BS_0.06%_ diet mediated an intestinal immune response in *S. aurata*, as we observed an upregulation of the cell marker cluster of differentiation 4-1 (*cd4-1*), a cell-surface marker of T lymphocytes, and other immune cells. This finding may not be directly explained by dietary BSs, but by their effect on shaping the gut microbiota, and particularly, by the increase in *Bacteroides*. As reported by Zhou and Zhi (2016), this Gram-negative genus has an immunomodulatory role due to its ability to induce the proliferation of regulatory CD4^+^ T cells (T_reg_) and production of anti-inflammatory cytokines. Additionally, the up-regulation of c*d4-1*, C-C chemokine receptor type 3 (*ccr3*), C-C chemokine receptor type 9 (*ccr9*), and C-C chemokine CK8 (*ck8*) may also support that fish fed the BS_0.06%_ had an enhanced gut immune response. This hypothesis would be in line with the study of Su et al. (2021), which reported an increase in the phagocytic and antibacterial activities in plasma of Pacific white shrimp (*Litopenaeus vannamei*) fed a diet supplemented with graded levels of a mixture of BSs (67.52% hyodeoxycholic acid, 19.81% chenodeoxycholic acid, and 8.60% hyocholic acid). Indeed, the cell marker CD4 can be found on the surface of T_reg_ cells, but also T helper cells, monocytes, macrophages, and dendritic cells (Parija, 2012; Ashfaq et al., 2019). These same immune cells, together with B cells, express the cell marker *ccr9*, which can function to induce the migration of immune cells to the gut to regulate inflammation (Pathak and Lal, 2020). There was also an up-regulation of *ccr3* which drives the displacement and activation of eosinophils (Heath et al., 1997), and of *ck8*, which elicits chemoattraction of lymphocytes and granulocytes (Hieshima et al., 1997) and can activate CCR6 (Baba et al., 1997), which is involved in differentiation of T cell-lineages during gut inflammation (Kulkarni et al., 2018). On the other hand, there was an up-regulation of galectin-1 (*lgals1*) and a down-regulation of galectin-8 (*lgals8*) and toll-like receptor 9 (*tlr9*), which may suggest the induction of an anti-inflammatory response in spite of the absence of changes in interleukin-10 (*il-10*) expression; although in the current study, the downregulation of *tlr9* and upregulation of *ck8* might be linked to the changes in bacterial abundance generated by the effect of BSs in the gut of *S. aurata* (Hemmi et al., 2000; Cuesta et al., 2010). In this sense, TLR9 recognizes bacterial unmethylated CpG motifs (Hemmi et al., 2000) and induces the production of some proinflammatory cytokines (Kumagai et al., 2008). Moreover, LGALS1 maintains the homeostasis of immune cells and the integrity of the mucosa and reduces inflammation by attenuating the synthesis of proinflammatory cytokines. It may also promote the apoptosis of T lymphocytes, the inactivation of antigen-presenting cells or the proliferation and differentiation of T cells (Seropian et al., 2018; Lu et al., 2019). Thus, the upregulation of *lgals1* may be in line with the reported up-regulation of *cd4-1* in fish fed the BS_0.06%_ diet. In contrast, we found a down-regulation in *lgals8* expression. LGALS8 favors inflammation by stimulating secretion of proinflammatory cytokines, including interleukin-6 (IL-6) and interleukin-8 (IL-8; Cattaneo et al., 2014). Thus, the hypothesis of a possible decrease in the exposure to potential pathogens due to the noxious effect of BSs might also be supported by the down-regulation of *lgals8*, considering that it can act as a sensor of membrane damage caused by infection and restricts the proliferation of infectious pathogens (Thurston et al., 2012). Although in the present study there was no variation in *il-6* expression, we found that *il-*8 was up-regulated in fish fed the BS_0.06%_ diet, and this may be associated to an increase in the expression of PRR macrophage mannose receptor 1 (*mrc1*; Gazi and Martinez-Pomares, 2009), providing evidence of an effective barrier against potential pathogens when no inflammatory disorders were found in gut (Gisbert et al., 2017). The former hypothesis may also be supported by the up-regulated expression of the membrane-bound form of immunoglobulin T (*igt-m*), widely used as a marker of mucosal immunocompetence (Zhang et al., 2010; Piazzon et al., 2016). The fact that IgM was not simultaneously up-regulated in fish fed BS_0.06%_ diet agrees with the recent studies that suggested differential regulatory mechanisms for *igt-m* and *igm* in *S. aurata* (Piazzon et al., 2016; Naya-Català et al., 2021). Considering that no inflammation was observed along the intestine of *S. aurata* fed diets supplemented with BSs (Ruiz et al., 2023), these results may indicate that dietary BS supplementation might have induced a state of immunocompetence in the intestine of *S. aurata* orchestrated by several genes related to a proinflammatory response with additional anti-inflammatory effectors, without having an overall apparent deleterious effect on gut physiology.

## 5. Conclusion

The present study showed that the supplementation of a high-saturated fat diet with a blend of BSs differentially modulated gut microbiota depending on the region in the intestine considered. In particular, dietary BSs decreased bacterial richness in the AI of *S. aurata*, but without remarkable changes in bacterial diversity and composition. Regarding the PI, dietary BSs had an impact on the relative abundance of some microbial taxa, resulting in an increase in the relative abundance of Firmicutes and a decrease of the phyla Proteobacteria, Actinobacteriota, and Campylobacterota, changes that were mainly linked to the fact that this region of the intestine is where the majority of primary BSs are metabolized into secondary BSs by bacterial enzymes. In addition, BSs had an antimicrobial effect on several Gram-negative bacteria, such as those from the genus *Corynebacterium*, *Sphingomonas*, and *Prevotella*. Dietary BSs promoted gut condition by mediating an intestinal immune response characterized by the regulation of several genes involved in innate and cellular immunity processes. Whether these changes are due to the immunogenic potential of BSs, by their role in shaping gut microbiota or both could not be deciphered; thus, they deserve further investigation.

## Data availability statement

The datasets presented in this study can be found in online repositories. The names of the repository/repositories and accession number(s) can be found at: NCBI—PRJNA915342.

## Ethics statement

The animal study was reviewed and approved by the Ethical Committee of the Institute for Food and Agriculture Research and Technology (IRTA), which adopts “The European Code of Conduct for Research Integrity,” and by the Generalitat of Catalunya (CEEA 219/2020).

## Author contributions

AR: methodology, formal analysis, visualization, and writing-original draft. KA: methodology, visualization, writing—review and editing, and supervision. DF and JC-G: methodology and writing—review and editing. PH: formal analysis and writing—review and editing. MV and JP-S: methodology, visualization, and writing—review and editing. EG: conceptualization, methodology, writing—review and editing, supervision, project administration, and funding acquisition. All authors contributed to the article and approved the submitted version.

## Funding

This work has been financed through the ADIPOQUIZ project (RTI2018-095653-R-I00) funded by the Ministerio de Ciencia, Innovación y Universidades (Spain). AR was supported by a predoctoral grant (PRE2019-091259) linked to the ADIPOQUIZ project.

## Conflict of interest

The authors declare that the research was conducted in the absence of any commercial or financial relationships that could be construed as a potential conflict of interest.

## Publisher’s note

All claims expressed in this article are solely those of the authors and do not necessarily represent those of their affiliated organizations, or those of the publisher, the editors and the reviewers. Any product that may be evaluated in this article, or claim that may be made by its manufacturer, is not guaranteed or endorsed by the publisher.

## References

[ref1] Al-Lahham S. H. Peppelenbosch M. P. Roelofsen H. Vonk R. J. Venema K. (2010). Biological effects of propionic acid in humans; metabolism, potential applications and underlying mechanisms. Biochim. Biophys. Acta 1801, 1175–1183. doi: 10.1016/j.bbalip.2010.07.007 20691280

[ref2] Ashfaq H. Soliman H. Saleh M. El-Matbouli M. (2019). CD4: a vital player in the teleost fish immune system. Vet. Res. 50, 1–11. doi: 10.1186/s13567-018-0620-0, PMID: 30616664 PMC6323851

[ref3] Baba M. Imai T. Nishimura M. Kakizaki M. Takagi S. Hieshima K.. (1997). Identification of CCR6, the specific receptor for a novel lymphocyte-directed CC chemokine LARC. J. Biol. Chem. 272, 14893–14898. doi: 10.1074/jbc.272.23.14893, PMID: 9169459

[ref4] Bakke A. M. Glover C. Krogdahl Å. (2010). “Feeding, digestion and absorption of nutrients” in Fish physiology: The multifunctional gut of fish. eds. Grosell M. Farrell A. P. Brauner C. J. (London: Academic Press), 57–110.

[ref5] Baya A. M. Lupiani B. Bandin I. Hetrick F. M. Figueras A. Carnanan A.. (1992). Phenotypic and pathobiological properties *of Corynebacterium aquaticum* isolated from diseased striped bass. Dis. Aquat. Organ. 14, 115–126. doi: 10.3354/dao014115

[ref6] Begley M. Gahan C. G. Hill C. (2005). The interaction between bacteria and bile. FEMS Microbiol. Rev. 29, 625–651. doi: 10.1016/j.femsre.2004.09.003 16102595

[ref7] Bray J. R. Curtis J. T. (1957). An ordination of the upland forest communities of southern Wisconsin. Ecol. Monogr. 27, 325–349. doi: 10.2307/1942268

[ref8] Broom L. J. Kogut M. H. (2018). The role of the gut microbiome in shaping the immune system of chickens. Vet. Immunol. Immunopathol. 204, 44–51. doi: 10.1016/j.vetimm.2018.10.002, PMID: 30596380

[ref9] Buddington R. K. Krogdahl A. Bakke-McKellep A. M. (1997). The intestines of carnivorous fish: structure and functions and the relations with diet. Acta Physiol. Scand. 638, 67–80. 9421581

[ref10] Caballero M. J. Obach A. Rosenlund G. Montero D. Gisvold M. Izquierdo M. S. (2002). Impact of different dietary lipid sources on growth, lipid digestibility, tissue fatty acid composition and histology of rainbow trout, *Oncorhynchus mykiss*. Aquaculture 214, 253–271. doi: 10.1016/S0044-8486(01)00852-3

[ref11] Callahan B. J. McMurdie P. J. Rosen M. J. Han A. W. Johnson A. J. A. Holmes S. P. (2016). DADA2: high-resolution sample inference from Illumina amplicon data. Nat. Methods 13, 581–583. doi: 10.1038/nmeth.3869, PMID: 27214047 PMC4927377

[ref12] Cattaneo V. Tribulatti M. V. Carabelli J. Carestia A. Schattner M. Campetella O. (2014). Galectin-8 elicits pro-inflammatory activities in the endothelium. Glycobiology 24, 966–973. doi: 10.1093/glycob/cwu060, PMID: 24957054

[ref13] Chattopadhyay I. Gundamaraju R. Jha N. K. Gupta P. K. Dey A. Mandal C. C.. (2022). Interplay between dysbiosis of gut microbiome, lipid metabolism, and tumorigenesis: can gut dysbiosis stand as a prognostic marker in cancer? Dis. Markers 2022:2941248. doi: 10.1155/2022/2941248, PMID: 35178126 PMC8847007

[ref14] Chen Y. R. Jing Q. L. Chen F. L. Zheng H. Chen L. D. Yang Z. C. (2021). *Desulfovibrio* is not always associated with adverse health effects in the Guangdong gut microbiome project. PeerJ 9:e12033. doi: 10.7717/peerj.12033, PMID: 34466295 PMC8380029

[ref15] Chiang J. Y. Ferrell J. M. (2022). Discovery of farnesoid X receptor and its role in bile acid metabolism. Mol. Cell. Endocrinol. 548:111618. doi: 10.1016/j.mce.2022.111618, PMID: 35283218 PMC9038687

[ref16] Cuesta A. Dios S. Figueras A. Novoa B. Esteban M. A. Meseguer J.. (2010). Identification of six novel CC chemokines in gilthead seabream (*Sparus aurata*) implicated in the antiviral immune response. Mol. Immunol. 47, 1235–1243. doi: 10.1016/j.molimm.2009.12.014 20096460

[ref17] Ding T. Xu N. Liu Y. Du J. Xiang X. Xu D.. (2020). Effect of dietary bile acid (BA) on the growth performance, body composition, antioxidant responses and expression of lipid metabolism-related genes of juvenile large yellow croaker (*Larimichthys crocea*) fed high-lipid diets. Aquaculture 518:734768. doi: 10.1016/j.aquaculture.2019.734768

[ref18] Drew M. D. Ogunkoya A. E. Janz D. M. Van Kessel A. G. (2007). Dietary influence of replacing fish meal and oil with canola protein concentrate and vegetable oils on growth performance, fatty acid composition and organochlorine residues in rainbow trout (*Oncorhynchus mykiss*). Aquaculture 267, 260–268. doi: 10.1016/j.aquaculture.2007.01.002

[ref19] Du Z. Y. Clouet P. Zheng W. H. Degrace P. Tian L. X. Liu Y. J. (2006). Biochemical hepatic alterations and body lipid composition in the herbivorous grass carp (*Ctenopharyngodon idella*) fed high-fat diets. Br. J. Nutr. 95, 905–915. doi: 10.1079/bjn20061733, PMID: 16611380

[ref20] Estruch G. Collado M. Peñaranda D. Tomás Vidal A. Jover Cerdá M. Pérez Martínez G.. (2015). Impact of fishmeal replacement in diets for gilthead sea bream (*Sparus aurata*) on the gastrointestinal microbiota determined by pyrosequencing the 16S rRNA gene. PLoS One 10:e0136389. doi: 10.1371/journal.pone.0136389, PMID: 26317431 PMC4552794

[ref21] Foey A. Picchietti S. (2014). “Immune defences of teleost fish” in Aquaculture nutrition: Gut health, probiotics and prebiotics. eds. Merrifield D. Ringø E. (Oxford: Wiley-Blackwell), 14–52.

[ref22] Fonseca-Madrigal J. Karalazos V. Campbell P. J. Bell J. G. Tocher D. R. (2005). Influence of dietary palm oil on growth, tissue fatty acid compositions, and fatty acid metabolism in liver and intestine in rainbow trout (*Oncorhynchus mykiss*). Aquacult. Nutr. 11, 241–250. doi: 10.1111/j.1365-2095.2005.00346.x

[ref23] Food and Agriculture Organization of the United Nations (FAO). (2022). The state of world fisheries and aquaculture 2022. Towards Blue Transformation. Rome: FAO.

[ref24] Gazi U. Martinez-Pomares L. (2009). Influence of the mannose receptor in host immune responses. Immunobiology 214, 554–561. doi: 10.1016/j.imbio.2008.11.004 19162368

[ref25] Gisbert E. Andree K. B. Quintela J. C. Calduch-Giner J. A. Ipharraguerre I. R. Pérez-Sánchez J. (2017). Olive oil bioactive compounds increase body weight, and improve gut health and integrity in gilthead sea bream (*Sparus aurata*). Br. J. Nutr. 117, 351–363. doi: 10.1017/S0007114517000228, PMID: 28245885

[ref26] Gu M. Bai N. Kortner T. M. (2017). Taurocholate supplementation attenuates the changes in growth performance, feed utilization, lipid digestion, liver abnormality and sterol metabolism in turbot (*Scophthalmus maximus*) fed high level of plant protein. Aquaculture 468, 597–604. doi: 10.1016/j.aquaculture.2016.11.022

[ref27] Hao W. L. Lee Y. K. (2004). Microflora of the gastrointestinal tract: a review. Methods Mol. Biol. 268, 491–502. doi: 10.1385/1-59259-766-1:491 15156063

[ref28] Heath H. Qin S. Rao P. Wu L. LaRosa G. Kassam N.. (1997). Chemokine receptor usage by human eosinophils. The importance of CCR3 demonstrated using an antagonistic monoclonal antibody. J. Clin. Invest. 99, 178–184. doi: 10.1172/JCI119145, PMID: 9005985 PMC507784

[ref29] Hemmi H. Takeuchi O. Kawai T. Kaisho T. Sato S. Sanjo H.. (2000). A toll-like receptor recognizes bacterial DNA. Nature 408, 740–745. doi: 10.1038/35047123 11130078

[ref30] Hieshima K. Imai T. Opdenakker G. van Damme J. Kusuda J. Tei H.. (1997). Molecular cloning of a novel human CC chemokine liver and activation-regulated chemokine (LARC) expressed in liver: chemotactic activity for lymphocytes and gene localization on chromosome 2. J. Biol. Chem. 272, 5846–5853. doi: 10.1074/jbc.272.9.5846, PMID: 9038201

[ref31] Hirano S. Masuda N. (1982). Enhancement of the 7 alpha-dehydroxylase activity of a gram-positive intestinal anaerobe by *Bacteroides* and its significance in the 7-dehydroxylation of ursodeoxycholic acid. J. Lipid Res. 23, 1152–1158. doi: 10.1016/S0022-2275(20)38052-4, PMID: 6960114

[ref32] Hofmann A. F. (1999). The continuing importance of bile acids in liver and intestinal disease. Arch. Intern. Med. 159, 2647–2658. doi: 10.1001/archinte.159.22.2647, PMID: 10597755

[ref33] Houten S. M. Volle D. H. Cummins C. L. Mangelsdorf D. J. Auwerx J. (2007). In vivo imaging of farnesoid X receptor activity reveals the ileum as the primary bile acid signaling tissue. Mol. Endocrinol. 21, 1312–1323. doi: 10.1210/me.2007-0113, PMID: 17426284

[ref34] Hu H. Shao W. Liu Q. Liu N. Wang Q. Xu J.. (2022). Gut microbiota promotes cholesterol gallstone formation by modulating bile acid composition and biliary cholesterol secretion. Nat. Commun. 13, 252–213. doi: 10.1038/s41467-021-27758-8, PMID: 35017486 PMC8752841

[ref35] Illumina. (2013). 16S metagenomic sequencing library preparation: preparing 16S ribosomal RNA gene amplicons for the Illumina MiSeq system. Available at: https://www.illumina.com/content/dam/illumina-support/documents/documentation/chemistry_documentation/16s/16s-metagenomic-library-prep-guide-15044223-b.pdf (accessed 11 November 2022).

[ref36] Islam K. S. Fukiya S. Hagio M. Fujii N. Ishizuka S. Ooka T.. (2011). Bile acid is a host factor that regulates the composition of the cecal microbiota in rats. Gastroenterology 141, 1773–1781. doi: 10.1053/j.gastro.2011.07.046, PMID: 21839040

[ref37] Iwashita Y. Suzuki N. Matsunari H. Sugita T. Yamamoto T. (2009). Influence of soya saponin, soya lectin, and cholyltaurine supplemented to a casein-based semipurified diet on intestinal morphology and biliary bile status in fingerling rainbow trout *Oncorhynchus mykiss*. Fish. Sci. 75, 1307–1315. doi: 10.1007/s12562-009-0158-1

[ref38] Iwashita Y. Suzuki N. Yamamoto T. Shibata J. I. Isokawa K. Soon A. H.. (2008). Supplemental effect of cholyltaurine and soybean lecithin to a soybean meal-based fish meal-free diet on hepatic and intestinal morphology of rainbow trout *Oncorhynchus mykiss*. Fish. Sci. 74, 1083–1095. doi: 10.1111/j.1444-2906.2008.01628.x

[ref39] Jiang M. Wen H. Gou G. W. Liu T. L. Lu X. Deng D. F. (2018). Preliminary study to evaluate the effects of dietary bile acids on growth performance and lipid metabolism of juvenile genetically improved farmed tilapia (*Oreochromis niloticus*) fed plant ingredient-based diets. Aquacult. Nutr. 24, 1175–1183. doi: 10.1111/anu.12656

[ref40] Jin M. Pan T. Cheng X. Zhu T. T. Sun P. Zhou F.. (2019). Effects of supplemental dietary L-carnitine and bile acids on growth performance, antioxidant and immune ability, histopathological changes and inflammatory response in juvenile black seabream (*Acanthopagrus schlegelii*) fed high-fat diet. Aquaculture 504, 199–209. doi: 10.1016/j.aquaculture.2019.01.063

[ref41] Kawamoto K. Horibe I. Uchida K. (1989). Purification and characterization of a new hydrolase for conjugated bile acids, chenodeoxycholyltaurine hydrolase, from *Bacteroides vulgatus*. J. Biochem. 106, 1049–1053. doi: 10.1093/oxfordjournals.jbchem.a122962, PMID: 2628421

[ref42] Keating N. Keely S. J. (2009). Bile acids in regulation of intestinal physiology. Curr. Gastroenterol. Rep. 11, 375–382. doi: 10.1007/s11894-009-0057-8 19765365

[ref43] Kim B. R. Shin J. Guevarra R. B. Lee J. H. Kim D. W. Seol K. H.. (2017). Deciphering diversity indices for a better understanding of microbial communities. J. Microbiol. Biotechnol. 27, 2089–2093. doi: 10.4014/jmb.1709.09027, PMID: 29032640

[ref44] Klindworth A. Pruesse E. Schweer T. Peplies J. Quast C. Horn M.. (2013). Evaluation of general 16S ribosomal RNA gene PCR primers for classical and next-generation sequencing-based diversity studies. Nucleic Acids Res. 41:e1. doi: 10.1093/nar/gks808, PMID: 22933715 PMC3592464

[ref45] Kong B. Wang L. Chiang J. Y. Zhang Y. Klaassen C. D. Guo G. L. (2012). Mechanism of tissue-specific farnesoid X receptor in suppressing the expression of genes in bile-acid synthesis in mice. Hepatology 56, 1034–1043. doi: 10.1002/hep.25740, PMID: 22467244 PMC3390456

[ref46] Kozińska A. Paździor E. Pękala A. Niemczuk W. (2014). *Acinetobacter johnsonii* and *Acinetobacter lwoffii*—the emerging fish pathogens. J. Vet. Res. 58, 193–199. doi: 10.2478/bvip-2014-0029

[ref47] Kulkarni N. Meitei H. T. Sonar S. A. Sharma P. K. Mujeeb V. R. Srivastava S.. (2018). CCR6 signaling inhibits suppressor function of induced-T_reg_ during gut inflammation. J. Autoimmun. 88, 121–130. doi: 10.1016/j.jaut.2017.10.013, PMID: 29126851

[ref48] Kumagai Y. Takeuchi O. Akira S. (2008). TLR9 as a key receptor for the recognition of DNA. Adv. Drug Deliv. Rev. 60, 795–804. doi: 10.1016/j.addr.2007.12.004 18262306

[ref49] Lallès J. P. (2020). Intestinal alkaline phosphatase in the gastrointestinal tract of fish: biology, ontogeny, and environmental and nutritional modulation. Rev. Aquac. 12, 555–581. doi: 10.1111/raq.12340

[ref50] Li X. Chen H. Guan Y. Li X. Lei L. Liu J.. (2013). Acetic acid activates the AMP-activated protein kinase signaling pathway to regulate lipid metabolism in bovine hepatocytes. PLoS One 8:e67880. doi: 10.1371/journal.pone.0067880, PMID: 23861826 PMC3701595

[ref51] Li Y. Wang S. Hu Y. Cheng J. Cheng X. Cheng P.. (2021). Dietary bile acid supplementation reveals beneficial effects on intestinal healthy status of tongue sole (*Cynoglossus semiliaevis*). Fish Shellfish Immunol. 116, 52–60. doi: 10.1016/j.fsi.2021.06.020, PMID: 34216786

[ref52] Lickwar C. R. Camp J. G. Weiser M. Cocchiaro J. L. Kingsley D. M. Furey T. S.. (2017). Genomic dissection of conserved transcriptional regulation in intestinal epithelial cells. PLoS Biol. 15:e2002054. doi: 10.1371/journal.pbio.2002054, PMID: 28850571 PMC5574553

[ref53] Liu C. Cui Y. Li X. Yao M. (2021). Microeco: an R package for data mining in microbial community ecology. FEMS Microbiol. Ecol. 97:fiaa255. doi: 10.1093/femsec/fiaa255 33332530

[ref54] Livak K. J. Schmittgen T. D. (2001). Analysis of relative gene expression data using real-time quantitative PCR and the 2^−ΔΔCT^ method. Methods 25, 402–408. doi: 10.1006/meth.2001.1262 11846609

[ref55] Lothong M. Tachampa K. Assavacheep P. Angkanaporn K. (2016). Effects of dietary betaine supplementation on back fat thickness and serum IGF-1 in late finishing pigs. Thai J. Vet. Med. 46, 427–434.

[ref56] Lu H. Dai X. Li X. Sun Y. Gao Y. Zhang C. (2019). Gal-1 regulates dendritic cells-induced Treg/Th17 balance though NF-κB/RelB-IL-27 pathway. Ann. Transl. Med. 7:628. doi: 10.21037/atm.2019.11.02, PMID: 31930029 PMC6944572

[ref57] Malick R. C. Bera A. K. Chowdhury H. Bhattacharya M. Abdulla T. Swain H. S.. (2020). Identification and pathogenicity study of emerging fish pathogens *Acinetobacter junii* and *Acinetobacter pittii* recovered from a disease outbreak in *Labeo catla* (Hamilton, 1822) and *Hypophthalmichthys molitrix* (Valenciennes, 1844) of freshwater wetland in West Bengal, India. Aquacult. Res. 51, 2410–2420. doi: 10.1111/are.14584

[ref58] Markandey M. Bajaj A. Ilott N. E. Kedia S. Travis S. Powrie F.. (2021). Gut microbiota: sculptors of the intestinal stem cell niche in health and inflammatory bowel disease. Gut Microbes 13:1990827. doi: 10.1080/19490976.2021.1990827, PMID: 34747326 PMC8583176

[ref59] McKnight D. T. Huerlimann R. Bower D. S. Schwarzkopf L. Alford R. A. Zenger K. R. (2019). Methods for normalizing microbiome data: an ecological perspective. Methods Ecol. Evol. 10, 389–400. doi: 10.1111/2041-210X.13115

[ref60] Nakanishi T. Shibasaki Y. Matsuura Y. (2015). T cells in fish. Biology 4, 640–663. doi: 10.3390/biology4040640, PMID: 26426066 PMC4690012

[ref61] Naya-Català F. do Vale Pereira G. Piazzon M. C. Fernandes A. M. Calduch-Giner J. A. Sitjà-Bobadilla A.. (2021). Cross-talk between intestinal microbiota and host gene expression in gilthead sea bream (*Sparus aurata*) juveniles: insights in fish feeds for increased circularity and resource utilization. Front. Physiol. 12:748265. doi: 10.3389/fphys.2021.748265, PMID: 34675821 PMC8523787

[ref62] Ng W. K. Sigholt T. Gordon Bell J. (2004). The influence of environmental temperature on the apparent nutrient and fatty acid digestibility in Atlantic salmon (*Salmo salar* L.) fed finishing diets containing different blends of fish oil, rapeseed oil and palm oil. Aquacult. Res. 35, 1228–1237. doi: 10.1111/j.1365-2109.2004.01131.x

[ref63] Olsen R. E. Myklebust R. Ringø E. Mayhew T. M. (2000). The influences of dietary linseed oil and saturated fatty acids on caecal enterocytes in Arctic char (*Salvelinus alpinus* L.): a quantitative ultrastructural study. Fish Physiol. Biochem. 22, 207–216. doi: 10.1023/A:1007879127182

[ref64] O’Neal C. C. Kohler C. C. (2008). Effect of replacing menhaden oil with catfish oil on the fatty acid composition of juvenile channel catfish, *Ictalurus punctatus*. J. World. Aquac. Soc. 39, 62–71. doi: 10.1111/j.1749-7345.2007.00137.x

[ref65] Parija S. C. (2012). Textbook of microbiology and immunology. India: Elsevier Health Sciences.

[ref66] Pathak M. Lal G. (2020). The regulatory function of CCR9^+^ dendritic cells in inflammation and autoimmunity. Front. Immunol. 11:536326. doi: 10.3389/fimmu.2020.536326, PMID: 33123124 PMC7566413

[ref67] Peng X. R. Feng L. Jiang W. D. Wu P. Liu Y. Jiang J.. (2019). Supplementation exogenous bile acid improved growth and intestinal immune function associated with NF-κB and TOR signalling pathways in on-growing grass carp (*Ctenopharyngodon idella*): enhancement the effect of protein-sparing by dietary lipid. Fish Shellfish Immunol. 92, 552–569. doi: 10.1016/j.fsi.2019.06.047, PMID: 31252043

[ref68] Pérez T. Balcázar J. L. Ruiz-Zarzuela I. Halaihel N. Vendrell D. De Blas I.. (2010). Host–microbiota interactions within the fish intestinal ecosystem. Mucosal Immunol. 3, 355–360. doi: 10.1038/mi.2010.12 20237466

[ref69] Piazzon M. C. Galindo-Villegas J. Pereiro P. Estensoro I. Calduch-Giner J. A. Gómez-Casado E.. (2016). Differential modulation of IgT and IgM upon parasitic, bacterial, viral, and dietary challenges in a perciform fish. Front. Immunol. 7:637. doi: 10.3389/fimmu.2016.00637, PMID: 28082977 PMC5186763

[ref70] Praslickova D. Torchia E. C. Sugiyama M. G. Magrane E. J. Zwicker B. L. Kolodzieyski L.. (2012). The ileal lipid binding protein is required for efficient absorption and transport of bile acids in the distal portion of the murine small intestine. PLoS One 7:e50810. doi: 10.1371/journal.pone.0050810, PMID: 23251388 PMC3519535

[ref71] Rader B. A. (2017). Alkaline phosphatase, an unconventional immune protein. Front. Immunol. 8:897. doi: 10.3389/fimmu.2017.00897, PMID: 28824625 PMC5540973

[ref72] Raschperger E. Thyberg J. Pettersson S. Philipson L. Fuxe J. Pettersson R. F. (2006). The coxsackie-and adenovirus receptor (CAR) is an in vivo marker for epithelial tight junctions, with a potential role in regulating permeability and tissue homeostasis. Exp. Cell Res. 312, 1566–1580. doi: 10.1016/j.yexcr.2006.01.025, PMID: 16542650

[ref73] Renga B. Mencarelli A. Cipriani S. D’Amore C. Carino A. Bruno A.. (2013). The bile acid sensor FXR is required for immune-regulatory activities of TLR-9 in intestinal inflammation. PLoS One 8:e54472. doi: 10.1371/journal.pone.0054472, PMID: 23372731 PMC3555871

[ref74] Ridlon J. M. Kang D. J. Hylemon P. B. Bajaj J. S. (2014). Bile acids and the gut microbiome. Curr. Opin. Gastroenterol. 30, 332–338. doi: 10.1097/MOG.0000000000000057, PMID: 24625896 PMC4215539

[ref75] Romano N. Kumar V. Yang G. Kajbaf K. Rubio M. B. Overturf K.. (2020). Bile acid metabolism in fish: disturbances caused by fishmeal alternatives and some mitigating effects from dietary bile inclusions. Rev. Aquac. 12, 1792–1817. doi: 10.1111/raq.12410

[ref76] Romero J. Ringø E. Merrifield D. (2014). “The gut microbiota of fish” in Aquaculture nutrition: Gut health, probiotics and prebiotics. eds. Merrifield D. Ringø E. (Oxford: Wiley-Blackwell), 75–100.

[ref77] Rueda-Jasso R. Conceiçao L. E. Dias J. De Coen W. Gomes E. Rees J. F.. (2004). Effect of dietary non-protein energy levels on condition and oxidative status of Senegalese sole (*Solea senegalensis*) juveniles. Aquaculture 231, 417–433. doi: 10.1016/S0044-8486(03)00537-4

[ref78] Ruiz A. Andree K. B. Sanahuja I. Holhorea P. G. Calduch-Giner J. A. Morais S.. (2023). Bile salt dietary supplementation promotes growth and reduces body adiposity in gilthead seabream (*Sparus aurata*). Aquaculture 566:739203. doi: 10.1016/j.aquaculture.2022.739203

[ref79] Salomón R. Firmino J. P. Reyes-López F. E. Andree K. B. González-Silvera D. Esteban M. A.. (2020). The growth promoting and immunomodulatory effects of a medicinal plant leaf extract obtained from *Salvia officinalis* and *Lippia citriodora* in gilthead seabream (*Sparus aurata*). Aquaculture 524:735291. doi: 10.1016/j.aquaculture.2020.735291

[ref80] Schubert K. Olde Damink S. W. von Bergen M. Schaap F. G. (2017). Interactions between bile salts, gut microbiota, and hepatic innate immunity. Immunol. Rev. 279, 23–35. doi: 10.1111/imr.12579, PMID: 28856736

[ref81] Seropian I. M. González G. E. Maller S. M. Berrocal D. H. Abbate A. Rabinovich G. A. (2018). Galectin-1 as an emerging mediator of cardiovascular inflammation: mechanisms and therapeutic opportunities. Mediators Inflamm. 2018:8696543. doi: 10.1155/2018/8696543, PMID: 30524200 PMC6247465

[ref82] Sinha J. Chen F. Miloh T. Burns R. C. Yu Z. Shneider B. L. (2008). β-Klotho and FGF-15/19 inhibit the apical sodium-dependent bile acid transporter in enterocytes and cholangiocytes. Am. J. Physiol. Gastrointest. Liver Physiol. 295, G996–G1003. doi: 10.1152/ajpgi.90343.2008, PMID: 18772362 PMC2584833

[ref83] Siyal F. A. Babazadeh D. Wang C. Arain M. A. Saeed M. Ayasan T.. (2017). Emulsifiers in the poultry industry. Worlds Poult. Sci. J. 73, 611–620. doi: 10.1017/S0043933917000502

[ref84] Smith P. E. Waters S. M. Gómez Expósito R. Smidt H. Carberry C. A. McCabe M. S. (2020). Synthetic sequencing standards: a guide to database choice for rumen microbiota amplicon sequencing analysis. Front. Microbiol. 11:606825. doi: 10.3389/fmicb.2020.606825, PMID: 33363527 PMC7752867

[ref85] Su C. Liu X. Li J. Zhang M. Pan L. Lu Y.. (2021). Effects of bile acids on the growth performance, lipid metabolism, non-specific immunity and intestinal microbiota of Pacific white shrimp (*Litopenaeus vannamei*). Aquacult. Nutr. 27, 2029–2041. doi: 10.1111/anu.13338

[ref86] Subhadra B. Lochmann R. Rawles S. Chen R. (2006). Effect of dietary lipid source on the growth, tissue composition and hematological parameters of largemouth bass (*Micropterus salmoides*). Aquaculture 255, 210–222. doi: 10.1016/j.aquaculture.2005.11.043

[ref87] Tamura A. Hayashi H. Imasato M. Yamazaki Y. Hagiwara A. Wada M.. (2011). Loss of claudin-15, but not claudin-2, causes Na^+^ deficiency and glucose malabsorption in mouse small intestine. Gastroenterology 140, 913–923. doi: 10.1053/j.gastro.2010.08.006, PMID: 20727355

[ref88] Tapia-Paniagua S. T. Balebona M. D. C. Firmino J. P. Rodríguez C. Polo J. Moriñigo M. A.. (2020). The effect of spray-dried porcine plasma on gilthead seabream (*Sparus aurata*) intestinal microbiota. Aquacult. Nutr. 26, 801–811. doi: 10.1111/anu.13039

[ref89] Thurston T. L. Wandel M. P. von Muhlinen N. Foeglein Á. Randow F. (2012). Galectin 8 targets damaged vesicles for autophagy to defend cells against bacterial invasion. Nature 482, 414–418. doi: 10.1038/nature10744, PMID: 22246324 PMC3343631

[ref90] Tocher D. R. Dick J. R. MacGlaughlin P. Bell J. G. (2006). Effect of diets enriched in Δ6 desaturated fatty acids (18: 3n− 6 and 18: 4n− 3), on growth, fatty acid composition and highly unsaturated fatty acid synthesis in two populations of Arctic charr (*Salvelinus alpinus* L.). Comp. Biochem. Physiol. B Biochem. Mol. Biol. 144, 245–253. doi: 10.1016/j.cbpb.2006.03.001 16630735

[ref91] Trushenski J. Rosenquist J. Gause B. (2011). Growth performance, tissue fatty acid composition, and consumer appeal of rainbow trout reared on feeds containing terrestrially derived rendered fats. N. Am. J. Aquac. 73, 468–478. doi: 10.1080/15222055.2011.633691

[ref92] Turchini G. M. Mentasti T. Frøyland L. Orban E. Caprino F. Moretti V. M.. (2003). Effects of alternative dietary lipid sources on performance, tissue chemical composition, mitochondrial fatty acid oxidation capabilities and sensory characteristics in brown trout (*Salmo trutta* L.). Aquaculture 225, 251–267. doi: 10.1016/S0044-8486(03)00294-1

[ref93] Vallejos-Vidal E. Reyes-Cerpa S. Tort L. Polo J. Reyes-López F. E. Gisbert E. (2022). Spray-dried porcine plasma promotes the association between metabolic and immunological processes at transcriptional level in gilthead sea bream (*Sparus aurata*) gut. Front. Mar. Sci. 9:814233. doi: 10.3389/fmars.2022.814233

[ref94] Vavassori P. Mencarelli A. Renga B. Distrutti E. Fiorucci S. (2009). The bile acid receptor FXR is a modulator of intestinal innate immunity. J. Immunol. 183, 6251–6261. doi: 10.4049/jimmunol.0803978 19864602

[ref95] Wada M. Tamura A. Takahashi N. Tsukita S. (2013). Loss of claudins 2 and 15 from mice causes defects in paracellular Na^+^ flow and nutrient transport in gut and leads to death from malnutrition. Gastroenterology 144, 369–380. doi: 10.1053/j.gastro.2012.10.035, PMID: 23089202

[ref96] Weinstein M. R. Litt M. Kertesz D. A. Wyper P. Rose D. Coulter M.. (1997). Invasive infections due to a fish pathogen, *streptococcus iniae*. N. Engl. J. Med. 337, 589–594. doi: 10.1056/NEJM199708283370902, PMID: 9271480

[ref97] Wen J. Mercado G. P. Volland A. Doden H. L. Lickwar C. R. Crooks T.. (2021). Fxr signaling and microbial metabolism of bile salts in the zebrafish intestine. Sci. Adv. 7:eabg1371. doi: 10.1126/sciadv.abg1371, PMID: 34301599 PMC8302129

[ref98] White J. R. Nagarajan N. Pop M. (2009). Statistical methods for detecting differentially abundant features in clinical metagenomic samples. PLoS Comput. Biol. 5:e1000352. doi: 10.1371/journal.pcbi.1000352, PMID: 19360128 PMC2661018

[ref99] Xiong F. Wu S.-G. Zhang J. Jakovlic I. Li W.-X. Zou H.. (2018). Dietary bile salt types influence the composition of biliary bile acids and gut microbiota in grass carp. Front. Microbiol. 9:2209. doi: 10.3389/fmicb.2018.02209, PMID: 30279683 PMC6154720

[ref100] Yamamoto T. Suzuki N. Furuita H. Sugita T. Tanaka N. Goto T. (2007). Supplemental effect of bile salts to soybean meal-based diet on growth and feed utilization of rainbow trout *Oncorhynchus mykiss*. Fish. Sci. 73, 123–131. doi: 10.1111/j.1444-2906.2007.01310.x

[ref101] Yin P. Xie S. Zhuang Z. He X. Tang X. Tian L.. (2021). Dietary supplementation of bile acid attenuate adverse effects of high-fat diet on growth performance, antioxidant ability, lipid accumulation and intestinal health in juvenile largemouth bass (*Micropterus salmoides*). Aquaculture 531:735864. doi: 10.1016/j.aquaculture.2020.735864

[ref102] Zhang Y. Feng H. Liang X. F. He S. Lan J. Li L. (2022). Dietary bile acids reduce liver lipid deposition via activating farnesoid X receptor, and improve gut health by regulating gut microbiota in Chinese perch (*Siniperca chuatsi*). Fish Shellfish Immunol. 121, 265–275. doi: 10.1016/j.fsi.2022.01.010, PMID: 35026410

[ref103] Zhang Y. A. Salinas I. Li J. Parra D. Bjork S. Xu Z.. (2010). IgT, a primitive immunoglobulin class specialized in mucosal immunity. Nat. Immunol. 11, 827–835. doi: 10.1038/ni.1913, PMID: 20676094 PMC3459821

[ref104] Zhou J. S. Chen H. J. Ji H. Shi X. C. Li X. X. Chen L. Q.. (2018). Effect of dietary bile acids on growth, body composition, lipid metabolism and microbiota in grass carp (*Ctenopharyngodon idella*). Aquacult. Nutr. 24, 802–813. doi: 10.1111/anu.12609

[ref105] Zhou Y. Zhi F. (2016). Lower level of *Bacteroides* in the gut microbiota is associated with inflammatory bowel disease: a meta-analysis. Biomed. Res. Int. 2016:5828959. doi: 10.1155/2016/5828959, PMID: 27999802 PMC5143693

