# Supplementary material for: Modulation of gut microbiota and intestinal immune response in gilthead seabream (Sparus aurata) by dietary bile salt supplementation
Source: Front Microbiol. 2023 Apr 24;14:1123716. doi: 10.3389/fmicb.2023.1123716 (PMC10166234; doi:10.3389/fmicb.2023.1123716)

Supplementary Material

Supplementary Table 1. Fatty acid composition (mg g lipid^{-1}) of the two experimental diets: a basal diet used as a control and the same diet supplemented with a blend of bile salts (BSs) at a dietary inclusion level of 0.06% ($BS_{0.06\%}$).

	**Control**	**BS0.06%**
Myristic acid (C14:0)	9.58 ± 0.37	8.61 ± 0.21
Pentadecylic acid (C15:0)	1.44 ± 0.09	1.56 ± 0.01
Palmitic acid (C16:0)	117.17 ± 2.46	116.79 ± 0.21
Stearic acid (C18:0)	31.57 ± 0.51	31.97 ± 0.71
Saturated fatty acids (SFAs)	163.4 ± 2.40	162.23 ± 0.34
Palmitoleic acid (C16:1 n-7)	26.49 ± 0.54	26.63 ± 0.81
Oleic acid (C18:1 n-9)	190.26 ± 4.18	193.51 ± 0.64
Eicosenoic acid (C20:1 n-9)	2.41 ± 0.10	2.30 ± 0.02
Monounsaturated fatty acids (MUFAs)	220.00 ± 4.39	223.66 ± 1.36
Linoleic acid (C18:2 n-6)	153.85 ± 0.04	159.58 ± 2.10
Gamma-linolenic acid (C18:3 n-6)	1.95 ± 0.29	1.88 ± 0.38
Arachidonic acid (C20:4 n-6)	4.34 ± 0.06	4.23 ± 0.28
n-6 Polyunsaturated fatty acids (n-6 PUFAs)	160.14 ± 0.39	165.69 ± 2.76
Alpha-linolenic acid (C18:3 n-3)	11.70 ± 0.41	11.95 ± 0.11
Stearidonic acid (C18:4 n-3)	2.26 ± 0.24	2.72 ± 0.48
Eicosatetraenoic acid (C20:4 n-3)	0.94 ± 0.03	0.87 ± 0.00
Eicosapentaenoic acid (C20:5 n-3)	24.14 ± 1.23	24.77 ± 0.13
Docosapentaenoic acid (C22:5 n-3)	3.25 ± 0.16	3.65 ± 0.00
Docosahexaenoic acid (C22:6 n-3)	14.29 ± 0.25	14.89 ± 0.11
n-3 Polyunsaturated fatty acids (n-3 PUFAs)	57.39 ± 2.33	59.53 ± 0.58
Total PUFAs	217.53 ± 2.71	225.23 ± 2.17

Supplementary Table 2. Gilthead seabream (*Sparus aurata*) primers for qPCR amplification of intestinal genes.

Gene	Symbol	GenBank	Primer
Proliferating cell nuclear antigen	*pcna*	KF857335	F: CGT ATC TGC CGT GAC CTG T<br>R: AGA ACT TGA CTC CGT CCT TGG
Transcription factor HES-1-B	*hes1-b*	KF857344	F: GCC TGC CGA TAT GAT GGA A<br>R: GGA GTT GTG TTC ATG CTT GC
Krüppel-like factor 4	*klf4*	KF857346	F: ACA TCA CCG CAC GCA CAC<br>R: AAC CAC AGC CCT CCC AGT C
Claudin-12	*cldn12*	KF861992	F: CTC TCA GGG CTA CAC ATC TAC CTA TGC<br>R: ACA TTC GTG AGC GGC TGG AG
Claudin-15	*cldn15*	KF861993	F: CCG ATT GTG GAA GTA GTG GCT CTG GT<br>R: CAG CAT CAC CCA ACC GAC GAA CC
Cadherin-1	*cdh1*	KF861995	F: TGC TCC ATA CAG CGT CAC CTT ACA<br>R: CTC GTT CAT CCT AGC CGT CCA GTT
Cadherin-17	*cdh17*	KF861996	F: GAT GCC CGC AAC CCA GAG<br>R: CCG TTG ATT CAC TGC CGT AGA C
Tight junction protein ZO-1	*tjp1*	KF861994	F: AAG CAG TAT TAC GGT GAC TCA<br>R: TGC ATC CCT GGC TTG TAG
Desmoplakin	*dsp*	KF861999	F: GCA GAA GGA GCA CGA GAC CATC<br>R: GGG TGT TCT TGT CGC AGG TGA A
Gap junction Cx32.2 protein	*cx32.2*	KF862000	F: CGA GGT GTT CTA TCT GCT CTG TA<br>R: CTT GTG GGT GCG AGT CCT
Coxsackievirus and adenovirus receptor homolog	*cxadr*	KF861998	F: CAT CAG AGG ACT ACG AGA GG<br>R: CAT CTT GGC AGC ATT TGG T
Intestinal-type alkaline phosphatase	*alpi*	KF857309	F: CCG CTA TGA GTT GGA CCG TGA T<br>R: GCT TTC TCC ACC ATC TCA GTA AGG G
Liver type fatty acid-binding protein	*fabp1*	KF857311	F: GTC CTC GTC AAC ACC TTC ACC AT<br>R: CGC CTT CAT CTT CTC GCC AGT
Intestinal fatty acid-binding protein	*fabp2*	KF857310	F: CGA GCA CAT TCC GCA CCA AAG<br>R: CCC ACG CAC CCG AGA CTT C
Ileal fatty acid-binding protein	*fabp6*	KF857312	F: ACC CAG GAC GGC AAT ACC<br>R: CGA CGG TGA AGT TGT TGG T
Mucin 2	*muc2*	JQ277710	F: ACG CTT CAG CAA TCG CAC CAT<br>R: CCA CAA CCA CAC TCC TCC ACA T
Mucin 13	*muc13*	JQ277713	F: TTC AAA CCC GTG TGG TCC AG<br>R: GCA CAA GCA GAC ATA GTT CGG ATA T
Tumor necrosis factor-alpha	*tnf-α*	AJ413189	F: CAG GCG TCG TTC AGA GTC TC<br>R: CTG TGG CTG AGA GCT GTG AG
Interleukin-1 beta	*il-1β*	AJ419178	F: GCG ACC TAC CTG CCA CCT ACA CC<br>R: TCG TCC ACC GCC TCC AGA TGC
Interleukin-6	*il-6*	EU244588	F: TCT TGA AGG TGG TGC TGG AAG TG<br>R: AAG GAC AAT CTG CTG GAA GTG AGG
Interleukin-7	*Il-7*	JX976618	F: CTA TCT CTG TCC CTG TCC TGT GA<br>R: TGC GGA TGG TTG CCT TGT AAT
Interleukin-8	*il-8*	JX976619	F: CAG CAG AGT CTT CAT CGT CAC TAT TG<br>R: AGG CTC GCT TCA CTG ATG G

Interleukin-10	*il-10*	JX976621	F: AAC ATC CTG GGC TTC TAT CTG<br>R: GTG TCC TCC GTC TCA TCT G
Interleukin-12 subunit beta	*il-12β*	JX976624	F: ATT CCC TGT GTG GTG GCT GCT<br>R: GCT GGC ATC CTG GCA CTG AAT
Interleukin-15	*il-15*	JX976625	F: GAG ACC AGC GAG CGA AAG GCA TCC<br>R: GCC AGA ACA GGT TAC AGG TTG ACA GGA A
Interleukin-34	*il-34*	JX976629	F: TCT GTC TGC CTG CTG GTA G<br>R: ATG CTG GCT GGT GTC TGG
CD4-1	*cd4-1*	AM489485	F: TCCTCCTCCTCGTCCTCGTT<br>R: GGTGTCTCATCTTCCGCTGTCT
CD8 beta	*cd8b*	KX231275	F: CCGAAATGTGGAAGACTGGAACTC<br>R: CTTTGGAGGTAAGGTTGGAGGGAT
C-C chemokine receptor type 3	*ccr3*	KF857317	F: CTA CAT CAG CAT CAC CAT ACG CAT CCT<br>R: TGG CAC GGC ACT TCT CCT TCA
C-C chemokine receptor type 9	*ccr9*	KF857318	F: TCC CTG AGT TAA TCT TCG CCC AAG TG<br>R: TGT TGT ATT CGT TGT TCC AGT AGA CCA GAG
C-C chemokine receptor type 11	*ccr11*	KF857319	F: GCT ACG ATT ACA GTT ATG AA<br>R: TAG ATG ATT GGG AGG AAG
C-C chemokine CK8 / C-C motif chemokine 20	*ck8/ ccl20*	GU181393	F: CCG TCC TCA TCT GCT TCA TAC T<br>R: GCT CTG CCG TTG ATG GAA C
Macrophage colony-stimulating factor 1 receptor 1	*csf1r1*	AM050293	F: TTG CGT GTG GTG AGG AAG GAA GGT<br>R: AGC AGG CAG GGC AGC AGG TA
Immunoglobulin M	*igm*	JQ811851	F: ACC TCA GCG TCC TTC AGT GTT TAT GAT GCC<br>R: CAG CGT CGT CGT CAA CAA GCC AAG C
Immunoglobulin T membrane-bound form	*igt-m*	KX599201	F: AGA CGA TGC CAG TGA AGA GGA TGA GT<br>R: CGA AGG AGG AGG CTG TGG ACC A
Galectin-1	*lgals1*	KF862003	F: GTG TGA GGA GGT CCG TGA TG<br>R: ACT GTA GAG CCG TCC GAT AGG
Galectin-8	*lgals8*	KF862004	F: GGC GGT GAA CGG CGG TCA<br>R: GCT CCA GCT CCA GTC TGT GTT GAT AC
Toll-like receptor 2	*tlr2*	KF857323	F: CAT CTG CGA CTC TCC TCT CTT CCT<br>R: ATT CAA CAA TGG AGC GGT GGA CTT
Toll-like receptor 5	*tlr5*	KF857324	F: TCG CCA ATC TGA CGG ACC TGA G<br>R: CAG AAC GCC GAT GTG GTT GTA AGA C
Toll-like receptor 9	*tlr9*	AY751797	F: GCC TTC CTT GTC TGC TCT TTC T<br>R: GCC GTA GAG GTG CTT CAG TAG
CD209 antigen-like protein D	*cd209d*	KF857327	F: CGC CAC GAG CAT GAG GAC AA<br>R: TCT TGC CAG AAT CCA TCA CCA TCC A
CD302 antigen	*cd302*	KF857328	F: GGA CCA GAG GAA GAG CAC ATC<br>R: GAC CAG GGC GGA CAT CAG
Macrophage mannose receptor 1	*mrc1*	KF857326	F: CTT CCG ACC GTA CCT GTA CCT ACT CA<br>R: CGA TTC CAG CCT TCC GCA CAC TTA
Fucolectin	*fcl*	KF857331	F: CCA TAC TGC TGA ACA GAC CAA CC<br>R: TGA TGG AGG TGA CGA TGT AGG A
β-Actin	*actb*	X89920	F: TCCTGCGGAATCCATGAGA<br>R: GACGTCGCACTTCATGATGCT

Supplementary Table 3. Mean ± SD and *P* values of the different alpha diversity indices, comparing among experimental groups with the Dunn's post-test (*P* < 0.05 in bold). Experimental groups (n = 12 fish per group): anterior (Control-AI) and posterior intestine (Control-PI) of gilthead seabream (*Sparus aurata*) fed the control diet, and anterior ($BS_{0.06\%}$-AI) and posterior intestine ($BS_{0.06\%}$-PI) of *S. aurata* fed a basal diet supplemented with a blend of bile salts at a dietary inclusion level of 0.06% ($BS_{0.06\%}$).

	**Control-AI**	**Control-PI**	**$BS_{0.06\%}$-AI**	**$BS_{0.06\%}$-PI**	$P_{\text{Control-AI vs BS0.06\%-AI}}$	$P_{\text{Control-PI vs BS0.06\%-PI}}$	$P_{\text{Control-AI vs Control-PI}}$	$P_{\text{BS0.06\%-AI vs BS0.06\%-PI}}$
Chao1	572.54 ± 189.53	493.17 ± 196.75	418.40 ± 114.96	370.18 ± 119.45	**0.048**	0.077	0.413	0.473
ACE	572.45 ± 189.35	493.18 ± 196.67	418.45 ± 115.02	370.21 ± 119.53	**0.048**	0.075	0.418	0.468
Shannon	5.661 ± 0.559	5.159 ± 1.027	5.567 ± 0.220	4.767 ± 1.392	0.428	0.479	0.052	0.063
Simpson	0.982 ± 0.037	0.963 ± 0.081	0.993 ± 0.002	0.896 ± 0.203	0.792	0.738	**0.016**	**0.024**

Supplementary Table 4. Mean ± SEM and *P* values of the relative abundances of the bacterial phyla from the intestine of gilthead seabream (*Sparus aurata*), comparing among experimental groups. (*Metastats*, $P < 0.05$). If $P < 0.1$ (in red bold), the divergences are considered as a tendency. Experimental groups (n = 12 fish per group): anterior (Control-AI) and posterior intestine (Control-PI) of *S. aurata* fed the control diet, and anterior ($BS_{0.06\%}$-AI) and posterior intestine ($BS_{0.06\%}$-PI) of *S. aurata* fed a basal diet supplemented with a blend of bile salts at a dietary inclusion level of 0.06% ($BS_{0.06\%}$).

	**Control-AI**	**Control-PI**	**$BS_{0.06\%}$-AI**	**$BS_{0.06\%}$-PI**	**$P_{Control-AI\ vs\ BS0.06\%-AI}$**	**$P_{Control-PI\ vs\ BS0.06\%-PI}$**	**$P_{Control-AI\ vs\ Control-PI}$**	**$P_{BS0.06\%-AI\ vs\ BS0.06\%-PI}$**
Firmicutes	31.17 ± 3.14	36.10 ± 2.66	36.75 ± 1.46	49.66 ± 4.85	0.253	**0.043**	0.142	**0.019**
Proteobacteria	29.31 ± 3.88	30.98 ± 5.92	21.96 ± 2.40	16.64 ± 1.87	0.236	**0.017**	0.267	0.103
Bacteroidota	19.43 ± 1.48	17.33 ± 2.27	21.15 ± 1.23	19.66 ± 2.44	0.594	0.206	0.227	0.379
Actinobacteriota	4.75 ± 0.43	4.92 ± 0.56	5.02 ± 0.31	3.50 ± 0.40	0.798	**0.067**	0.267	**0.021**
Desulfobacterota	1.85 ± 0.40	2.26 ± 0.70	4.06 ± 0.56	2.42 ± 0.51	**0.014**	0.256	0.258	**0.096**
Unassigned	3.95 ± 0.67	1.62 ± 0.31	2.44 ± 0.33	1.76 ± 0.25	0.149	0.237	**0.011**	0.121
Campylobacterota	1.56 ± 0.25	2.20 ± 0.37	1.58 ± 0.29	1.29 ± 0.26	1.000	**0.067**	0.142	0.325
Verrucomicrobiota	1.92 ± 0.38	1.03 ± 0.20	1.36 ± 0.25	0.99 ± 0.17	0.350	0.256	**0.065**	0.185
Chloroflexi	1.49 ± 0.61	0.42 ± 0.07	1.43 ± 0.32	0.83 ± 0.30	1.000	0.205	**0.026**	0.181

Supplementary Table 5. Mean ± SEM and *P* values of the relative abundances of the bacterial genera from the intestine of gilthead seabream (*Sparus aurata*), comparing among experimental groups (*Metastats*, $P < 0.05$). If $P < 0.1$ (in red bold), the divergences are considered as a tendency. Experimental groups (n = 12 fish per group): anterior (Control-AI) and posterior intestine (Control-PI) of *S. aurata* fed the control diet, and anterior ($BS_{0.06\%}$-AI) and posterior intestine ($BS_{0.06\%}$-PI) of *S. aurata* fed a basal diet supplemented with a blend of bile salts at a dietary inclusion level of 0.06% ($BS_{0.06\%}$).

	**Control-AI**	**Control-PI**	**$BS_{0.06\%}$-AI**	**$BS_{0.06\%}$-PI**	$P_{\text{control-AI vs BS0.06\%-AI}}$	$P_{\text{control-PI vs BS0.06\%-PI}}$	$P_{\text{control-AI vs Control-PI}}$	$P_{\text{BS0.06\%-AI vs BS0.06\%-PI}}$
Firmicutes|Clostridia|Lachnospirales|Lachnospiraceae|Unassigned	4.60 ± 0.88	4.32 ± 0.81	6.78 ± 0.75	5.59 ± 1.19	**0.037**	0.189	0.215	0.141
Bacteroidota|Bacteroidia|Bacteroidales|Bacteroidaceae|*Bacteroides*	2.21 ± 0.41	2.66 ± 0.47	4.09 ± 0.59	4.69 ± 1.62	**0.010**	0.156	0.162	0.187
Bacteroidota|Bacteroidia|Bacteroidales|Muribaculaceae|Unassigned	2.50 ± 0.41	2.56 ± 0.54	4.25 ± 0.73	3.51 ± 0.56	**0.026**	0.132	0.237	0.141
Firmicutes|Bacilli|Lactobacillales|Streptococcaceae|*Streptococcus*	1.47 ± 0.31	1.20 ± 0.23	0.91 ± 0.17	8.92 ± 8.05	**0.066**	0.292	0.173	0.148
Firmicutes|Clostridia|Peptostreptococcales-Tissierellales|Family XI|*Fenollaria*	2.68 ± 0.64	3.74 ± 0.87	1.85 ± 0.50	1.69 ± 0.59	0.129	**0.053**	0.134	0.191
Firmicutes|Clostridia|Clostridiales|Clostridiaceae|*Candidatus* Arthromitus	0.02 ± 0.02	2.38 ± 1.47	0.03 ± 0.03	8.54 ± 5.73	0.160	0.174	**0.008**	**< 0.001**
Unassigned|Unassigned|Unassigned|Unassigned|Unassigned	3.95 ± 0.67	1.62 ± 0.31	2.44 ± 0.33	1.76 ± 0.27	**0.031**	0.269	**0.006**	**0.065**
Proteobacteria|Gammaproteobacteria|Pseudomonadales|Pseudomonadaceae|*Pseudomonas*	2.64 ± 0.54	2.83 ± 0.76	2.50 ± 0.44	1.53 ± 0.57	0.232	0.131	0.218	**0.082**
Bacteroidota|Bacteroidia|Bacteroidales|Porphyromonadaceae|*Porphyromonas*	2.02 ± 0.46	2.92 ± 0.61	1.52 ± 0.40	1.18 ± 0.33	0.156	**0.019**	0.106	0.148
Proteobacteria|Gammaproteobacteria|Pseudomonadales|Moraxellaceae|*Acinetobacter*	1.61 ± 0.36	2.52 ± 0.76	2.04 ± 0.53	1.03 ± 0.38	0.167	**0.074**	0.115	**0.068**
Bacteroidota|Bacteroidia|Flavobacteriales|Weeksellaceae|Unassigned	1.52 ± 0.36	1.55 ± 0.32	2.58 ± 0.31	1.48 ± 0.40	**0.018**	0.296	0.237	**0.026**
Firmicutes|Clostridia|Peptostreptococcales-Tissierellales|Family XI|*Ezakiella*	2.03 ± 0.51	2.17 ± 0.56	1.58 ± 0.43	1.07 ± 0.33	0.165	**0.072**	0.225	0.131
Proteobacteria|Gammaproteobacteria|Enterobacterales|Vibrionaceae|*Catenococcus*	0.18 ± 0.18	6.64 ± 5.21	0.00 ± 0.00	0.11 ± 0.11	0.160	0.101	0.104	0.141
Desulfobacterota|Desulfovibrionia|Desulfovibrionales|Desulfovibrionaceae|*Desulfovibrio*	1.20 ± 0.29	1.33 ± 0.41	2.44 ± 0.42	1.41 ± 0.36	**0.015**	0.296	0.214	**0.043**
Campylobacterota|Campylobacteria|Campylobacterales|Campylobacteraceae|*Campylobacter*	1.23 ± 0.28	1.82 ± 0.40	1.10 ± 0.30	0.80 ± 0.23	0.216	**0.037**	0.106	0.141
Actinobacteriota|Actinobacteria|Corynebacteriales|Corynebacteriaceae|*Corynebacterium*	1.16 ± 0.26	1.71 ± 0.28	1.10 ± 0.21	0.74 ± 0.21	0.240	**0.014**	**0.083**	**0.090**
Proteobacteria|Alphaproteobacteria|Caulobacterales|Caulobacteraceae|*Brevundimonas*	0.95 ± 0.13	1.10 ± 0.32	1.46 ± 0.15	1.05 ± 0.31	**0.010**	0.296	0.206	**0.093**
Proteobacteria|Alphaproteobacteria|Rhodobacterales|Rhodobacteraceae|*Marivita*	3.65 ± 3.07	0.14 ± 0.08	0.30 ± 0.11	0.13 ± 0.06	0.147	0.303	**0.085**	**0.082**
Proteobacteria|Gammaproteobacteria|Burkholderiales|Burkholderiaceae|*Ralstonia*	0.84 ± 0.11	1.25 ± 0.24	1.31 ± 0.22	0.95 ± 0.28	**0.030**	0.213	**0.071**	0.122
Proteobacteria|Alphaproteobacteria|Sphingomonadales|Sphingomonadaceae|*Sphingomonas*	1.09 ± 0.27	1.27 ± 0.27	1.08 ± 0.28	0.61 ± 0.14	0.255	**0.032**	0.194	**0.079**
Bacteroidota|Bacteroidia|Bacteroidales|Prevotellaceae|*Prevotella*	1.09 ± 0.16	1.23 ± 0.18	1.06 ± 0.17	0.66 ± 0.25	0.239	**0.053**	0.175	**0.082**
Firmicutes|Clostridia|Peptostreptococcales-Tissierellales|Family XI|*Peptoniphilus*	0.92 ± 0.21	1.62 ± 0.32	0.78 ± 0.23	0.78 ± 0.24	0.203	**0.041**	**0.043**	0.216

Supplementary Table 6. Relative gene expression of intestinal mRNA transcripts of 48 h fasted- and 2 h postprandial- gilthead seabream (*Sparus aurata*) that were fed with a control and a basal diet supplemented with a blend of bile salts at a dietary inclusion of 0.06 ($BS_{0.06\%}$). Values are the mean ± SEM of 8 fish. All data are in reference to the expression level of *hes1-b* in fed fish from control group with an arbitrary value of 1. Bold values indicate statistically significant differences ($P < 0.1$) in Student's t-test and two-way ANOVA.

	2 h postprandial (Student's t-test)			48 h fasted (Student's t-test)			Two-way ANOVA (*P*)		
	Control	$BS_{0.06\%}$	*P*	Control	$BS_{0.06\%}$	*P*	Diet	Nut. Status	Diet x Nut. Status
pcna	7.41 ± 0.39	6.30 ± 0.42	**0.071**	3.18 ± 0.50	3.97 ± 0.57	0.317	0.737	**<0.001**	**0.055**
hes1-b	1.03 ± 0.10	0.94 ± 0.14	0.584	0.99 ± 0.15	1.15 ± 0.12	0.416	0.818	0.529	0.328
klf4	2.63 ± 0.46	2.07 ± 0.28	0.307	1.32 ± 0.23	1.10 ± 0.07	0.408	0.207	**<0.001**	0.571
cldn12	0.40 ± 0.03	0.38 ± 0.04	0.705	0.42 ± 0.04	0.49 ± 0.03	0.12	0.371	**0.044**	0.158
cldn15	13.73 ± 0.77	11.88 ± 0.89	0.138	19.19 ± 1.26	23.94 ± 1.57	**0.033**	0.224	**<0.001**	**0.008**
cdh1	6.55 ± 0.46	5.62 ± 0.36	0.132	6.12 ± 0.42	7.25 ± 0.68	0.181	0.84	0.236	**0.049**
cdh17	31.29 ± 1.61	24.87 ± 1.28	**0.007**	33.14 ± 4.26	32.11 ± 1.90	0.828	0.155	**0.085**	0.299
tjp1	0.39 ± 0.02	0.38 ± 0.04	0.96	0.43 ± 0.10	0.36 ± 0.03	0.506	0.529	0.903	0.558
dsp	2.89 ± 0.24	2.85 ± 0.21	0.88	3.63 ± 0.33	3.50 ± 0.18	0.735	0.716	**0.01**	0.864
cx32.2	20.12 ± 2.65	13.21 ± 2.25	**0.067**	42.70 ± 6.68	43.75 ± 3.71	0.893	0.49	**<0.001**	0.351
cxadr	1.51 ± 0.06	1.45 ± 0.12	0.652	2.31 ± 0.14	2.81 ± 0.18	**0.047**	0.111	**<0.001**	**0.044**
alpi	21.76 ± 1.91	9.85 ± 1.25	**<0.001**	41.77 ± 4.97	52.53 ± 5.30	0.164	0.878	**<0.001**	**0.005**
fabp1	38.22 ± 1.59	36.33 ± 4.35	0.689	51.91 ± 4.89	51.90 ± 4.66	0.998	0.818	**0.001**	0.82
fabp2	207.10 ± 44.47	275.61 ± 43.38	0.289	443.90 ± 68.08	303.65 ± 48.43	0.126	0.5	**0.018**	**0.057**
fabp6	0.12 ± 0.02	87.73 ± 56.31	**0.089**	0.02 ± 0.00	0.03 ± 0.00	0.64	**0.053**	**0.052**	**0.053**
muc2	16.64 ± 1.42	15.36 ± 1.43	0.538	21.45 ± 3.57	18.25 ± 1.70	0.432	0.322	**0.093**	0.668
muc13	28.04 ± 1.77	22.29 ± 2.37	**0.08**	35.77 ± 4.23	35.62 ± 2.22	0.974	0.313	**0.001**	0.339
tnf-α	0.20 ± 0.03	0.18 ± 0.02	0.637	0.14 ± 0.01	0.14 ± 0.01	0.943	0.689	**0.027**	0.644
il-1β	0.09 ± 0.01	0.11 ± 0.01	0.356	0.06 ± 0.00	0.07 ± 0.01	0.233	0.164	**0.002**	0.736
il-6	0.14 ± 0.02	0.10 ± 0.02	0.211	0.05 ± 0.01	0.04 ± 0.00	0.123	0.102	**<0.001**	0.406
il-7	0.47 ± 0.05	0.36 ± 0.04	0.113	0.31 ± 0.03	0.39 ± 0.03	0.102	0.738	**0.096**	**0.023**
il-8	0.14 ± 0.01	0.18 ± 0.03	**0.086**	0.18 ± 0.03	0.22 ± 0.02	0.338	**0.095**	0.147	0.926
il-10	0.15 ± 0.02	0.14 ± 0.03	0.781	0.08 ± 0.01	0.07 ± 0.01	0.219	0.537	**<0.001**	0.938
il-12β	0.35 ± 0.03	0.34 ± 0.04	0.885	0.28 ± 0.04	0.27 ± 0.02	0.694	0.712	**0.028**	0.883
il-15	0.19 ± 0.01	0.17 ± 0.01	0.15	0.33 ± 0.04	0.28 ± 0.02	0.328	0.184	**<0.001**	0.554
il-34	0.56 ± 0.04	0.59 ± 0.05	0.683	1.05 ± 0.06	1.00 ± 0.07	0.577	0.842	**<0.001**	0.49
cd4-1	0.13 ± 0.01	0.19 ± 0.03	**0.034**	0.23 ± 0.02	0.20 ± 0.02	0.226	0.397	**0.008**	**0.015**
cd8b	0.04 ± 0.01	0.04 ± 0.01	0.912	0.06 ± 0.01	0.03 ± 0.00	**0.013**	**0.071**	0.936	**0.051**
ccr3	0.36 ± 0.02	0.47 ± 0.06	**0.097**	0.55 ± 0.05	0.57 ± 0.04	0.754	0.15	**0.004**	0.313
ccr9	0.51 ± 0.04	0.79 ± 0.07	**0.003**	1.60 ± 0.20	1.32 ± 0.06	0.21	0.996	**<0.001**	**0.014**
ccr11	1.45 ± 0.12	1.15 ± 0.16	0.169	3.09 ± 0.26	2.55 ± 0.23	0.147	**0.053**	**<0.001**	0.573
ck8 / ccl20	1.71 ± 0.22	3.04 ± 0.45	**0.024**	3.80 ± 0.52	4.29 ± 0.91	0.645	0.14	**0.01**	0.492
csf1r1	0.41 ± 0.04	0.56 ± 0.09	0.152	0.48 ± 0.06	0.51 ± 0.03	0.632	0.136	0.894	0.326
igm	4.68 ± 0.99	5.84 ± 1.19	0.468	7.31 ± 1.17	5.16 ± 1.43	0.27	0.687	0.429	0.184
igt-m	0.27 ± 0.04	0.39 ± 0.05	**0.062**	0.30 ± 0.03	0.30 ± 0.02	0.875	**0.085**	0.388	0.118
lgals1	6.20 ± 0.30	11.24 ± 1.91	**0.02**	7.92 ± 0.67	7.17 ± 0.57	0.404	**0.053**	0.278	**0.011**
lgals8	2.15 ± 0.16	1.62 ± 0.10	**0.015**	2.07 ± 0.22	2.38 ± 0.22	0.321	0.567	**0.07**	**0.027**
tlr2	0.28 ± 0.04	0.31 ± 0.04	0.498	0.44 ± 0.03	0.40 ± 0.02	0.332	0.897	**<0.001**	0.15
tlr5	0.10 ± 0.01	0.09 ± 0.02	0.516	0.04 ± 0.00	0.04 ± 0.00	0.518	0.42	**<0.001**	0.644
tlr9	0.128 ± 0.016	0.081 ± 0.013	**0.06**	0.053 ± 0.007	0.049 ± 0.003	0.619	**0.039**	**<0.001**	**0.078**
cd209d	0.14 ± 0.01	0.16 ± 0.01	0.337	0.19 ± 0.01	0.18 ± 0.02	0.74	0.771	**0.011**	0.414
cd302	3.68 ± 0.18	3.67 ± 0.26	0.96	5.93 ± 0.28	6.59 ± 0.40	0.196	0.278	**<0.001**	0.255
mrc1	0.59 ± 0.04	0.86 ± 0.13	**0.066**	0.96 ± 0.11	0.98 ± 0.08	0.86	0.145	**0.015**	0.225
fcl	0.62 ± 0.15	0.76 ± 0.30	0.682	3.77 ± 0.81	4.69 ± 1.61	0.603	0.508	**<0.001**	0.625

Supplementary Figure 1. Average forward and reverse read quality score plots. The green line represents the mean between all template quality scores; the continuous orange line, the median; and the discontinuous, the 25th and 75th quantiles. The grey scale heatmap represented the frequency of quality scores (higher in the darker zones). Samples come from the anterior (AI) and posterior intestine (PI) of gilthead seabream (*Sparus aurata*) fed a control and the experimental diet supplemented with a blend of bile salts at a dietary inclusion level of 0.06% ($BS_{0.06\%}$).

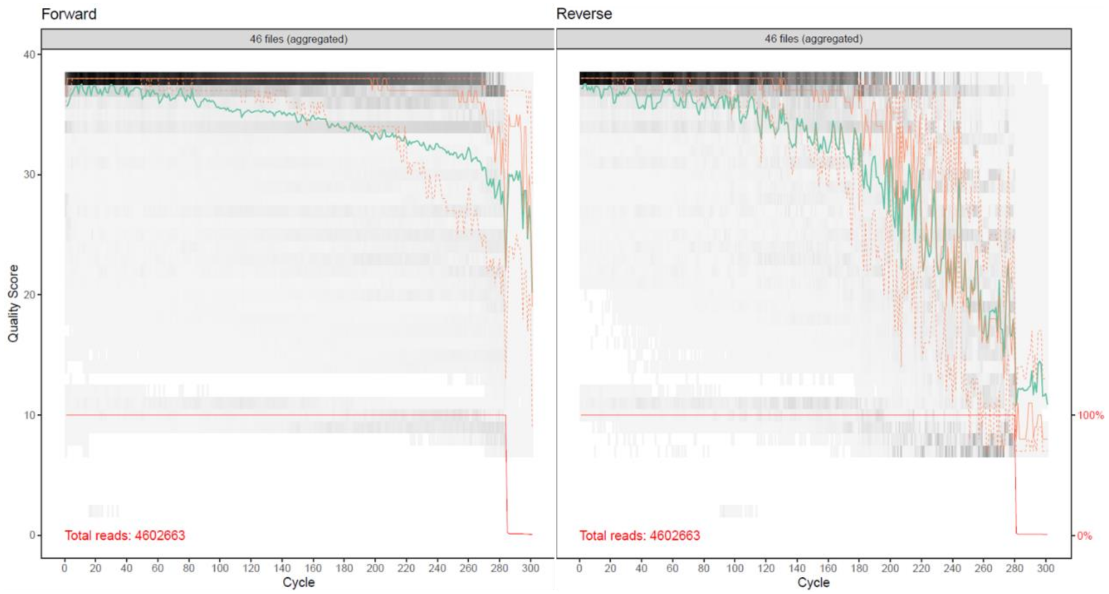

Supplementary Figure 2. Template rarefaction curves represented in function of observed ASVs from the anterior (AI) and posterior intestine (PI) of gilthead seabream (*Sparus aurata*) fed the control and the experimental diet supplemented with a blend of bile salts at a dietary inclusion level of 0.06% ($BS_{0.06\%}$).

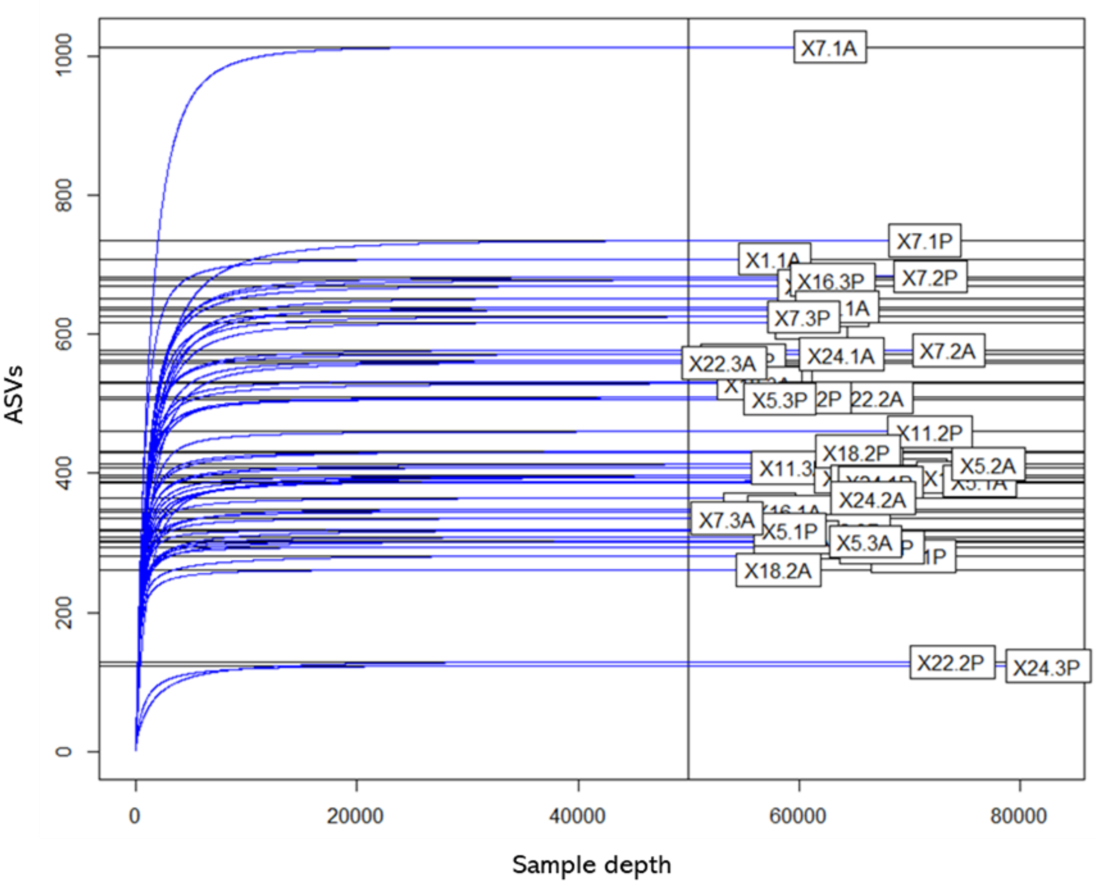

Supplementary Figure 3. Principal Coordinate Analysis (PCoA) plot of gilthead seabream (*Sparus aurata*) microbiota based on the Bray-Curtis dissimilarity. Experimental groups (n = 12 fish per group): anterior (Control-AI) and posterior intestine (Control-PI) of *S. aurata* fed the control diet, and anterior ($BS_{0.06\%}$-AI) and posterior intestine ($BS_{0.06\%}$-PI) of *S. aurata* fed a basal diet supplemented with a blend of bile salts at a dietary inclusion level of 0.06% ($BS_{0.06\%}$).

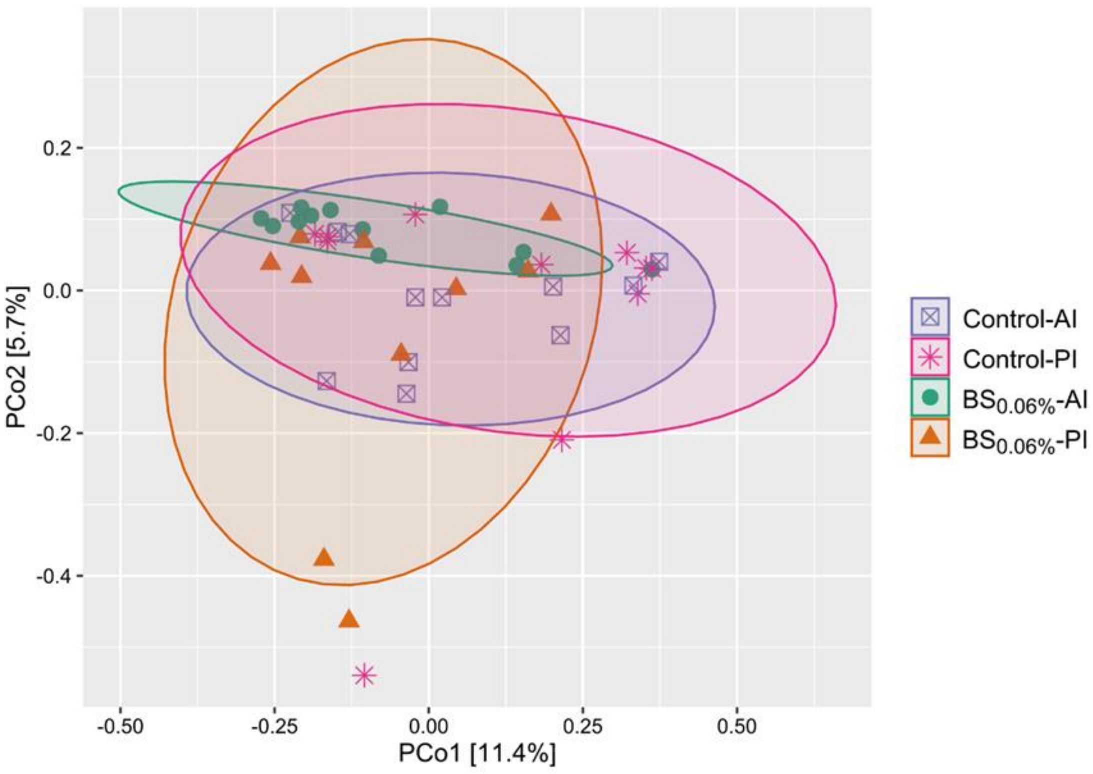

Supplementary Figure 4. Validation (permutation test) of the PLS-DA model constructed for analysing distribution of expression of biomarkers on anterior intestine (AI) from 48 h fasted- and 2 h postprandial- gilthead seabream (*Sparus aurata*; n = 8 per group) that were fed the control and the diet supplemented with bile salts at a dietary inclusion level of 0.06% ($BS_{0.06\%}$).

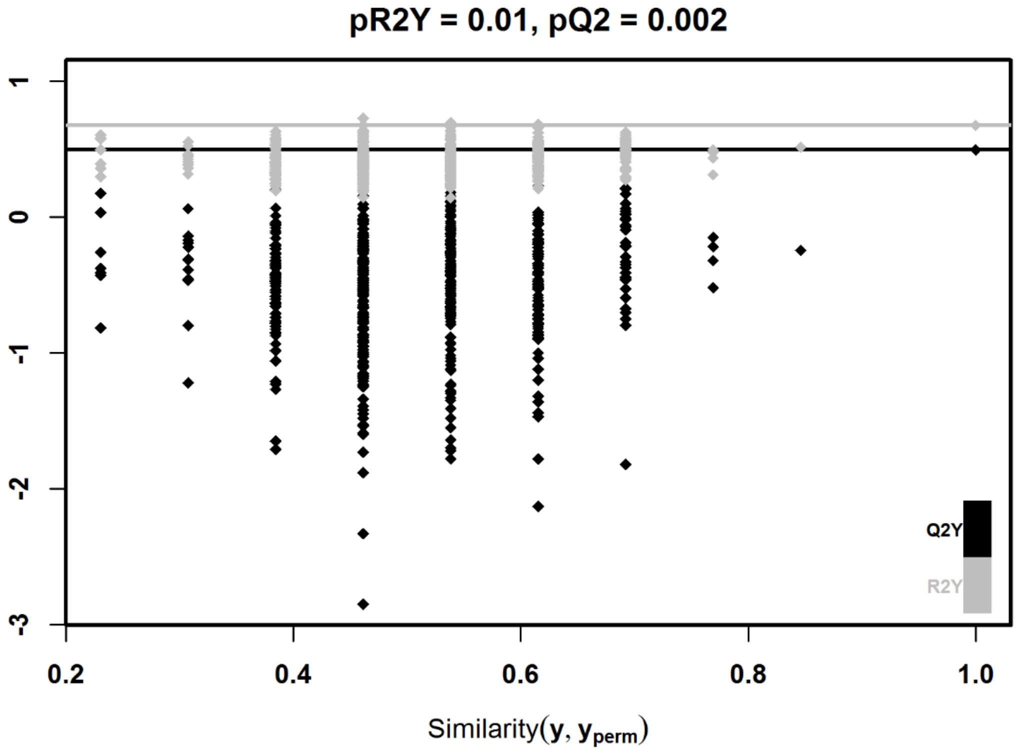

Supplement: Supplementary file 1 [file Data_Sheet_1.PDF]
